# The Limited Role of Number of Nested Syntactic Dependencies in Accounting for Processing Cost: Evidence from German Simplex and Complex Verbal Clusters

**DOI:** 10.3389/fpsyg.2017.02268

**Published:** 2018-01-23

**Authors:** Markus Bader

**Affiliations:** Department of Linguistics, Goethe University Frankfurt, Frankfurt am Main, Germany

**Keywords:** syntactic dependencies, processing complexity, center embedding, recursion, verb cluster, German

## Abstract

This paper presents three acceptability experiments investigating German verb-final clauses in order to explore possible sources of sentence complexity during human parsing. The point of departure was De Vries et al.'s (2011) generalization that sentences with three or more crossed or nested dependencies are too complex for being processed by the human parsing mechanism without difficulties. This generalization is partially based on findings from Bach et al. (1986) concerning the acceptability of complex verb clusters in German and Dutch. The first experiment tests this generalization by comparing two sentence types: (i) sentences with three nested dependencies within a single clause that contains three verbs in a complex verb cluster; (ii) sentences with four nested dependencies distributed across two embedded clauses, one center-embedded within the other, each containing a two-verb cluster. The results show that sentences with four nested dependencies are judged as acceptable as control sentences with only two nested dependencies, whereas sentences with three nested dependencies are judged as only marginally acceptable. This argues against De Vries et al.'s (2011) claim that the human parser can process no more than two nested dependencies. The results are used to refine the Verb-Cluster Complexity Hypothesis of Bader and Schmid (2009a). The second and the third experiment investigate sentences with four nested dependencies in more detail in order to explore alternative sources of sentence complexity: the number of predicted heads to be held in working memory (storage cost in terms of the Dependency Locality Theory [DLT], Gibson, 2000) and the length of the involved dependencies (integration cost in terms of the DLT). Experiment 2 investigates sentences for which storage cost and integration cost make conflicting predictions. The results show that storage cost outweighs integration cost. Experiment 3 shows that increasing integration cost in sentences with two degrees of center embedding leads to decreased acceptability. Taken together, the results argue in favor of a multifactorial account of the limitations on center embedding in natural languages.

## 1. Introduction

One of the few features of natural languages for which there is general agreement is the existence of non-local dependencies (Tallerman et al., 2009). Within psycholinguistics, non-local dependencies play a key role in several theories of the human parser, including the Syntactic Prediction Locality Theory (Gibson, 1998), the Dependency Locality Theory (Gibson, 2000), the Efficiency Theory (Hawkins, 2004, 2014), and the Minimize Dependencies Theory (Temperley, 2007; Gildea and Temperley, 2010). These theories have focused on two properties of syntactic dependencies, their number and their length, but syntactic dependencies have other properties which may be relevant too. The way dependencies are ordered is such a property, as pointed out by De Vries et al. (2011). Two overlapping dependencies can be ordered in one of three ways, as shown in (1), where D_n_ is dependent on H_(n)_.

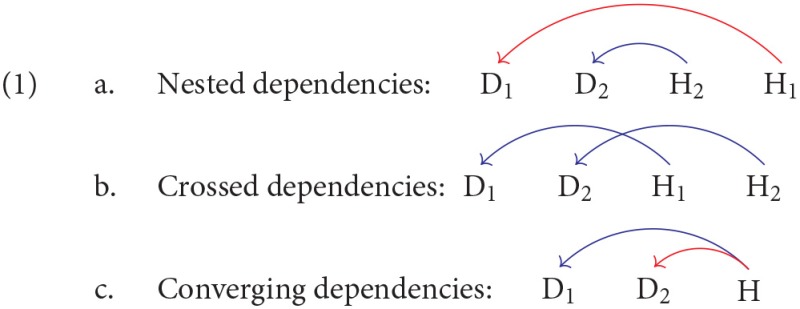

First, one dependency can be nested within the other, as in (1-a); second, two dependencies can cross each other, as in (1-b); third, two dependencies can converge on a single head, as in (1-c).

It has often been observed that sentences with multiple center embedding are difficult or even impossible to comprehend (see reviews in Gibson, 1998; De Vries et al., 2011). Even sentences with only two levels of center embedding and thus three nested dependencies, as illustrated in (2), can be difficult for the human parser to process. The reasons for this limitation on human parsing are still a matter of active research.

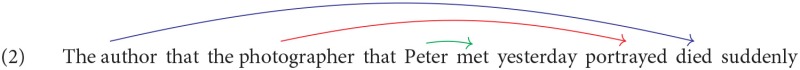

Sentences with doubly center embedded relative clauses are the most prominent instance of multiply nested dependencies, but they are not the only ones. As pointed out by De Vries et al. (2011), another instance is provided by sentences with certain types of complex verb clusters as they are found in the West-Germanic verb-final languages, including Dutch and German. Experimental evidence on this issue comes from a seminal study on crossed and nested dependencies by Bach et al. (1986). Bach et al. (1986) capitalized on the fact that Dutch and German, despite being syntactically highly similar, differ with regard to the order of verbs. When several verbs appear in a row in clause-final position, they form a so-called *verb cluster*. As illustrated in Table 1, verb clusters give rise to crossed dependencies in Dutch but nested dependencies in German.

**Table 1:**
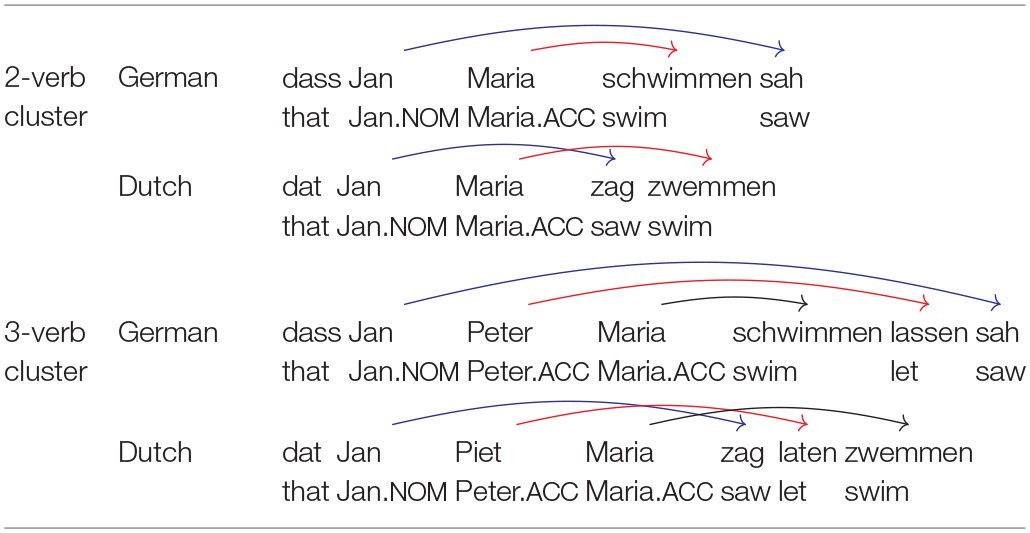
Order of dependencies in Dutch and German sentences with 2 and 3 verb clusters.

Table 1 shows only a subset of all dependencies in the sentences under consideration. The dependencies that are shown are those between verbs and their NP arguments, that is, those dependencies which are necessary for assigning semantic roles. For example, the NP *Jan* is the subject argument of *sag/sah* (“saw”). The dependencies between a verb and its verbal arguments, for example between *sag/sah* (“saw”) and *zwemmen/schwimmen* (“swim”) in the 2-verb cluster sentences, are not shown because these are all local dependencies which do not contribute to the issue of how nested dependencies affect sentence complexity.

Bach et al. (1986) had speakers of Dutch and German rate sentences as shown in Table 1 in their respective language. Verb clusters of size one to four were included in the study. Bach et al.'s (1986) experiment yielded two major results. First, sentences with two nested or crossed dependencies showed only a small decrease in acceptability in comparison to sentences with only a single dependency, but adding a third dependency caused acceptability to decline sharply. Going from two verbs to three verbs decreased acceptability by about three points on a scale from 1 to 10, and going from three verbs to four verbs led to a further decrease of 2 points. Order of dependencies did not have a significant effect for two dependencies, but for three or four dependencies, acceptability declined less sharply for crossed than for nested dependencies. On average, an advantage of about 0.4 points was observed when comparing crossed dependencies to nested dependencies for clusters of equal size. In sum, clusters of size three or greater are hardly processable whether they involve crossed or nested dependencies, and the disadvantage is somewhat stronger for nested than for crossed dependencies.

Based on Bach et al.'s (1986) finding as well as on evidence concerning multiply center-embedded relative clauses, De Vries et al. (2011) arrive at the conclusion that …

[…], since humans possess finite brains that are constrained by (among other things) memory limitations, we have problems comprehending and producing sentences with three or more nested or crossed dependencies […]

(De Vries et al., 2011, p. 12)

De Vries et al. (2011) put forward an interesting generalization which delimits the class of sentences leading to processing overload in an empirically testable way. In order to test this hypothesis and to explore the role that dependency formation may play for sentence complexity more generally, this paper presents three experiments that have investigated German verb-final clauses of varying complexity. The first experiment provides a test of De Vries et al.'s (2011) generalization. Since the results of this test show that the generalization is not correct, two further experiments explore alternative sources of sentence complexity, namely integration and storage cost as defined in the Dependency Locality Theory of Gibson (2000). Before the experiments are presented, the next section gives a short introduction to current accounts of parsing complexity.

## 2. Determinants of syntactic complexity

Memory and expectations are the main ingredients of current theories of syntactic processing complexity (see Nakatani and Gibson, 2008; Jaeger and Tily, 2011; Levy, 2013). With regard to memory, syntactic dependency formation during on-line sentence comprehension poses several requirements. When the two elements of a dependency relation occur adjacent to each other, the first element is still in the focus of attention and thus immediately available for being integrated with the second element. In the case of non-local dependencies, however, the first and second element of a dependency are separated by intervening material. In this case, the element of the dependency that comes first in the word string must be kept in working memory for later retrieval on encountering the second element. Keeping elements in memory and retrieving elements from memory are both possible sources of sentence complexity. The idea that parsing complexity varies with the number of dependencies for which the first element has already been encountered but not the second element, has been termed the *Incomplete Dependency Hypothesis* in Nakatani and Gibson (2008). This hypothesis is given in (3).

(3) *Incomplete Dependency Hypothesis*
The human sentence processor is sensitive to the number of partially processed dependencies at each processing
state.

For the case of converging dependencies, that is, dependencies in which several phrases are dependent on a single head, research on verb-final languages has repeatedly shown that increasing the number of incomplete dependencies does not lead to increased processing load and can make a sentence even less difficult to process. For example, Nakatani and Gibson (2008) ran a self-paced reading experiment investigating Japanese sentences with one degree of center embedding and a varying number of incomplete dependencies. Two conditions from this experiment are illustrated in (4).

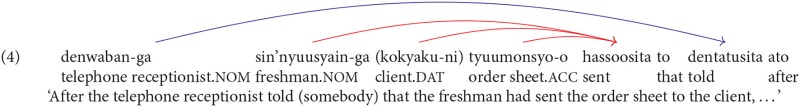

In (4), the higher temporal clause contains a subject and a complement clause. This complement clause in turn contains a subject, an accusative object, and optionally a dative object. Directly before encountering the verb of the embedded complement clause, there are three incomplete dependencies when the dative object is absent (the subject of the matrix clause and the subject and accusative object of the embedded clause) and four when the dative object is present. Despite the high number of incomplete dependencies, such sentences do not pose problems for the human parser and reading times were in fact lower in the presence of a dative object, that is, with four instead of three incomplete dependencies. Thus, instead of making sentences difficult to comprehend, a high number of incomplete dependencies can ease sentence processing. This effect, which was first found by Konieczny (2000), has become known as the *anti-locality effect* (see Vasishth and Lewis, 2006; Levy and Keller, 2013, for related findings).

In sum, there is abundant evidence showing that increasing the number of incomplete converging dependencies does not increase processing load. For nested and crossed dependencies, the situation is less clear. A close relationship between the number of nested dependencies and parsing complexity is suggested by sentences with multiple center embedding. As illustrated by example (2), sentences with two levels of center embedding contain three nested dependencies, and such sentences are difficult to process. Similar considerations hold for verb clusters with three or more verbs as investigated by Bach et al. (1986). Findings of this kind have led De Vries et al. (2011) to the generalization that processing three or more nested or crossed dependencies is beyond the normal capacity of the human parser.

As already pointed out above, non-local dependencies require not only to keep the first element in memory until the second element is encountered. They also require to retrieve the first element from the ongoing memory representation on encountering the second element. Retrieval may be difficult because the two elements are separated by intervening material. Of particular importance in this regard is the distance between the two elements of a dependency, that is, the length of the dependency. This aspect of dependency formation has been termed the *Bottom-up Head-dependent Distance Hypothesis* by Nakatani and Gibson (2008). This hypothesis is given in (5).

(5) *Bottom-up Head-dependent Distance Hypothesis*
The difficulty of integrating a new word w into the current structure depends on the distance back to the head h to
which w connects.

The distance between the two elements of a dependency can be measured in different ways. For the following discussion, dependency length is measured in terms of integration cost as proposed in the Dependency Locality Theory of Gibson (2000). The (total) integration cost of a word is the sum of its referential integration cost and its structural integration cost. Each word triggering the introduction of a new discourse referent is assumed to incur a referential integration cost of one unit, where nouns and verbs introduce new discourse referents and all other words do not (see Warren and Gibson, 2002, for a more fine-grained measure). Structural integration cost arises when a dependency has to be formed, that is, when a new input word must be integrated with a word already contained within sentence memory. Structural integration cost is a function of dependency length, with length measured in terms of the number of new discourse referents that intervene between the two items of a dependency.

Assigning a syntactic structure to a sentence during parsing involves more than computing the various dependencies that obtain between the words and phrases of the sentence. In particular, the human parser also has to compute a phrase-structure representation. This task provides a possible further source of sentence complexity. As long as the phrase-structure representation is not complete, the parser may form expectations about how the partial phrase-structure tree computed for the input string seen so far will be completed by the remainder of the input string. As in the case of incomplete dependencies, the simplest way to link phrase-structure expectations to sentence complexity is by counting the number of expectations that have to be held in working memory at each point during the ongoing parse. One implementation of this idea, which goes back to the early work of Yngve (1960) on language production, is stated in the *Predicted Syntactic Head Hypothesis* of Nakatani and Gibson (2008) given in (6).

(6) *Predicted Syntactic Head Hypothesis*
The human sentence processor is sensitive to the number of syntactic heads that are required to form a grammatical sentence at each processing
state.

In accordance with the DLT's notion of storage cost, it is assumed below that each predicted head is associated with one memory unit. In the following, only the storage cost associated with predicted verbal heads is considered, because storage cost related to other heads is always matched across sentences compared to each other.

To summarize, number of nested or crossed dependencies, the length of the dependencies, and the ongoing phrase-structure representation have been proposed as potential sources of sentence complexity. These sources do not exclude each other and they are not meant as an exhaustive list. The following three experiments were designed to test the contribution of each of the three sources of complexity. Whether the number of nested dependencies affects sentence complexity is the topic of Experiment 1. Experiment 2 tests the relative importance of integration cost and storage cost in sentences where the two make divergent predictions. The final Experiment 3 investigates the role of integration cost in sentences matched for number of nested dependencies and storage cost.

## 3. Experiment 1

The aim of Experiment 1 was to test De Vries et al.'s (2011) hypothesis that sentences with three or more crossed or nested dependencies cause problems for the human parsing mechanism. Experiment 1 tests this hypothesis by disentangling number of nested dependencies and verb cluster complexity. Verb cluster formation is a typologically rare property of the Germanic OV languages (see Wurmbrand, 2006, 2017, for comprehensive overviews). As documented by Wurmbrand (2006), syntactic analyses of verb clusters differ in many important ways from each other (see Seuren and Kempen, 2003, for a selection of verb cluster analysis within a broad range of syntactic frameworks). In the current context, the most important property of verb clusters is that in many respects, they behave like a single verbal head, independent of the number of verbs they contain. Thus, a sentence with a 3-verb cluster would get a surface structure along the lines of the mono-clausal representation in (7)^1^. Such a mono-clausal structure may be syntactically derived from a multi-clausal structure as in (8), but it can also be generated directly and without reference to any kind of multi-clausal syntactic structure. A multi-clausal representation may still be necessary, but only at the semantic level.

(7) Mono-clausal analysis of verb clusters
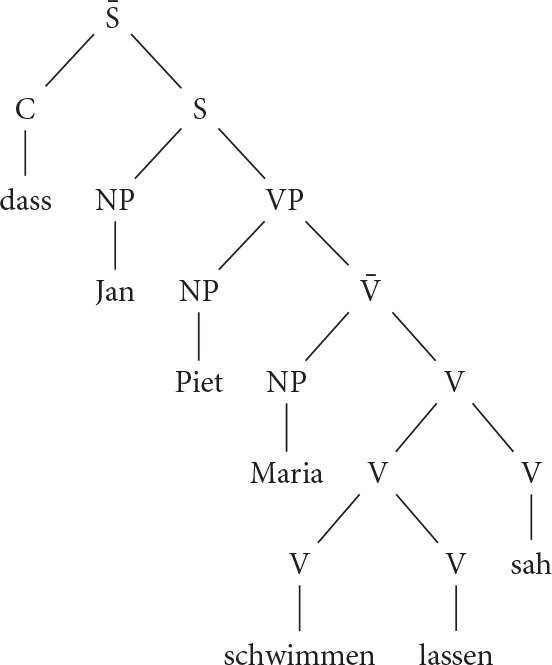

(8) Multi-clausal analysis of verb clusters
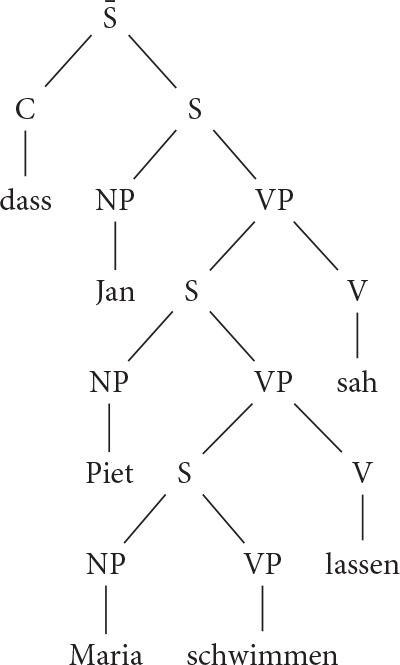

Verb clusters with three or more verbs in a row are not necessarily hard to process for the human parser. When only a single verb introduces arguments into the clause, verb clusters up to five verbs can be comprehended without much difficulty, and verb clusters of this size occur in authentic texts, as shown in (9) (see Bader and Schmid, 2009b; Bader et al., 2009, for experimental evidence and corpus data).

(9) … was  alles besser **hätte**_1_ gemacht_5_ worden_4_ sein_3_
          what all    better  had      mad         been      be
     können_2_
     can
     ‘what could have been made better’
     (www.dradio.de/dkultur/sendungen/fazit/2028303/)

With regard to the relationship between verb-cluster formation and sentence complexity, the empirical data can be summarized as follows. The data of Bach et al. (1986) indicate that verb clusters in which each verb introduces its own argument(s) are easy to process as long as no more than two verbs are involved. With three or more verbs, such clusters become difficult or even impossible to comprehend. Adding further verbs not introducing arguments of their own, in contrast, increases complexity only marginally if at all.

A possible source of the processing complexity observed in the case of Bach et al.'s (1986) sentences is verb-cluster formation itself. A proposal to this effect has been made by Bader and Schmid (2009a), based on an investigation of so-called *long passivization*, as illustrated by the example in (10).

(10) Es wurde berichtet, dass der          alte Vater
       [zu entlasten versucht] wurde.
       it   was     reported  that  the.nom  old  father
       to disburden tried        was
       ‘It was reported that one had tried to disburden the old
       father.’

Here the control verb *versuchen* (“to try”) occurs in the passive voice, as shown by its appearance as a past participle. The unexpected property of this construction is that the major change brought about by passivization, the promotion of the direct object to subject, does not affect the object of the passivized verb *versuchen* (“try”), but the object of the infinitival verb *zu entlasten* (“to disburden”), which is the complement of *versuchen* (“to try”). This object occurs with nominative case in (10) instead of accusative case, as it would in a corresponding active clause. Passivization thus has a kind of long-distance effect in this construction, hence the name long-distance passivization. However, if *zu entlasten* (“to disburden”) and *versuchen* (“to try”) form a verb cluster and thus a single complex predicate, passivization applies in the usual way. What is passivized is not *versuchen* (“to try”) itself, but *zu entlasten versuchen* (“to try to disburden”) as a whole. As shown by the somewhat reduced acceptability of this construction, forming a complex predicate and then applying passivization to it is not cost-free. Bader and Schmid (2009a) have therefore proposed the *Verb-Cluster Complexity Hypothesis* given in (11).

(11) *Verb-Cluster Complexity Hypothesis*
The argument-structure operations involved in verb-
  cluster formation are costly for the HSPM [= Human Sentence Processing Mechanism].

The Verb-Cluster Complexity Hypothesis was stated under the assumption that only verbs that have arguments of their own come with an argument structure. These are all lexical verbs, whereas functional verbs like auxiliaries and modals have no arguments. A small number of verbs have a hybrid status, like the verb *lassen* (“to let”), which has a causer argument but shows the syntactic behavior of modal verbs. Under these assumptions, the Verb-Cluster Complexity Hypothesis distinguishes between verb clusters that involve only a single verb with an argument structure and verb clusters in which the argument structures of several verbs must be combined in some way. What this hypothesis does not predict is why there is a rather sharp decline in acceptability when more than two argument structures must be combined, as in the 3- and 4-verb clusters investigated by Bach et al. (1986).

A major drawback of the *Verb-Cluster Complexity Hypothesis* is that it is specifically tailored to the case of verb-cluster formation. This contrasts with De Vries et al.'s (2011) account, which derives the complexity observed for clusters with three or more verbs from a general constraint on human parsing, namely that parsing proceeds smoothly only when a sentence contains no more than two crossed or nested dependencies. This generalization predicts that three or more nested dependencies should cause high processing complexity independently of whether a complex verb cluster is involved or not. This prediction can be tested with the help of sentences in which three or more nested dependencies are distributed across several verb clusters with at most two verbs. Two examples with three nested dependencies distributed across two verb clusters—a 1-verb cluster and a 2-verb cluster—are shown in (12).

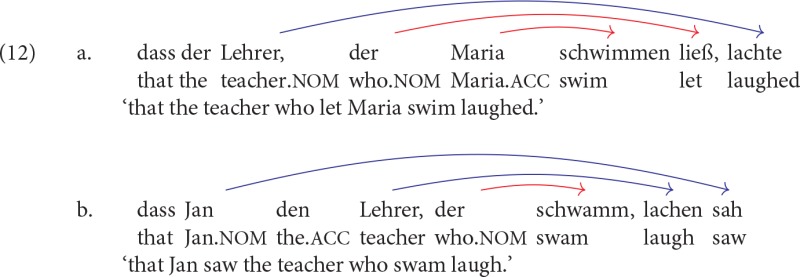

When both the upper and the lower clause contain a 2-verb cluster, four nested dependencies result, as shown in (13).

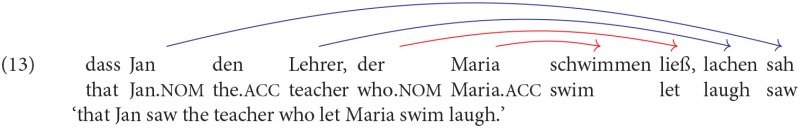

If it were true that sentences with three or more nested dependencies exceed the normal capacity of the human parsing mechanism, sentences as in (12) and (13) should be at least as difficult to process than the three- and four-verb cluster sentences investigated by Bach et al. (1986). If, on the other hand, the findings of Bach et al. (1986) reflect processing complexity tied to verb-cluster formation itself, then sentences containing three or more nested dependencies should become easier to process when they do not contain a complex verb cluster. These predictions are tested in Experiment 1 by comparing the complexity of sentences containing three nested dependences originating in a single 3-verb cluster (1×3 sentences) to the complexity of sentences with four nested dependencies distributed across two verb clusters with two verbs each (2×2 sentences). Complexity will be assessed using an acceptability rating task instead of an on-line measure in order to obtain results that are comparable to the results of Bach et al. (1986).

1×3 sentences are structurally similar to the 3-verb cluster sentences investigated by Bach et al. (1986). Given their results, 1×3 sentences are expected to be of marginal acceptability. If De Vries et al. (2011) are correct and it is the presence of three nested dependencies which makes 1×3 sentences difficult to comprehend, then 2×2 sentences, which contain four nested dependencies, should be even less acceptable. If, on the other hand, the complexity of 1×3 sentences is intimately tied to the presence of a 3-verb cluster, 2×2 sentences should be more acceptable than 1×3 sentences.

### 3.1. Methods

#### 3.1.1. Participants

Sixty-four students from the Goethe-University Frankfurt completed a questionnaire for course credit. All participants were native speakers of German and naive with respect to the purpose of the experiment. Ethical approval was not required for this study in accordance with the national and institutional guidelines.

#### 3.1.2. Materials

Sixteen sentences were constructed for Experiment 1. Each sentence appeared in four versions according to the two factors Dependencies (1×3 vs. 2×2) and Structure (center embedded vs. control). Center embedded 1×3 sentences were included to replicate the finding that three-verb clusters as investigated by Bach et al. (1986) are difficult to comprehend. Bach et al. are not very explicit concerning their experimental material and give only an example sentence representing their three-verb cluster condition. This sentence is reproduced in (14).

(14) Arnim         hat Wolfgang         der        Lehrerin
       Arnim.nom has Wolfgang.acc  the.dat teacher
       die        Murmeln aufräumen helfen lassen.
       the.acc marbels   collect-up  help    let
       Arnim let Wolfgang help the teacher collect up the
       marbles.

In addition to containing a complex verb cluster, sentence (14) is complex in several other ways. First, because this sentence is a main clause, a composite tense form with the finite auxiliary in the verb-second position must be used in order to have three verbs in clause-final position. Since for some of the verbs used by Bach et al. (1986) there was an uncertainty with regard to the morphological form required in the perfect tense (past participle or infinitive), the authors ran two subexperiments varying the morphological form. Second, this sentence is more complicated than the sentences considered so far because it contains four arguments instead of three, which is a consequence of using the control verb *helfen* (“to help”), which has a dative object in addition to its verbal complement. Third, *helfen* (“to help”) can also be used with a *zu* (“to”) infinitive instead of a bare infinitive, introducing some indeterminacy that is not found with other verbs selecting a verbal complement.

Since these complications may have contributed to the reduced acceptability of sentences with complex verb clusters in Bach et al. (1986), Experiment 1 investigated center embedded 1×3 sentences that differed in several ways from sentences as in (14). First of all, the complex verb cluster was contained within an embedded verb-final clause in Experiment 1. As a consequence, the finite verb appeared clause-finally and it was no longer necessary to use a composite tense form. Instead, all verb clusters ended with a main verb in the past tense. Second, only three verbs selecting a verbal complement were used. The hierarchically highest and thus the finite verb was always the verb *sah* (“saw”), which is the most frequent perception verb. This verb selected either the verb *lassen* (“let”) or the verb *versuchen* (“try”), which is the most acceptable control verb in this kind of construction (Schmid et al., 2005). All three verbs unambiguously determine the morphological form of their verbal complement.

Using the three verbs *sah* (“saw”), *lassen* (“let”) and *versuchen* (“try”), three types of sentences were constructed. All three sentence types instantiate the structural pattern described in the introduction to Experiment 1, but differ in several item-specific ways. If the complexity of these sentences in the four different experimental conditions is mainly driven by structural factors, all three sentence types are expected to show the same pattern of acceptability. Should Experiment 1 reveal distinct acceptability patterns for the three sentence types, the assumption that complexity is a function of sentence structure would have to be abandoned.

An example sentence for each of the three sentence types is shown in Table 2; the complete sentence set is available as Supplementary Material. All sentences consisted of the main clause “Ich weiß” (“I know”) followed by a *that*-clause. In the condition “center-embedded with 1×3 dependencies,” the *that*-clause contained three NPs followed by a 3-verb cluster. The first NP was a proper name and the second and third NP were definite NPs marked for accusative case. Eight sentences contained a 3-verb cluster of the form “lexical verb – *lassen* (“let”) – *sah* (“saw”)”. In four of the sentences with *lassen*, the lexical verb was intransitive and the third NP realized the subject argument of this verb, as in the example discussed above. In the other four sentences with *lassen*, the lexical verb was a transitive verb and its object was realized by the third NP. In this case, the subject of the lexical verb is implicitly understood as “someone.”

**Table 2:** Three complete stimulus sentences from Experiment 1, one with a causative verb and one with a control verb.

**CAUSATIVE VERB *LASSEN* TO LET—INTRANSITIVE COMPLEMENT**		
1 × 3	center	dass Moritz den Gärtner den Kunden warten lassen sah.
	embedded	that M.nom the.acc gardener the.acc costumer wait let see
		‘that Moritz saw the gardener letting the costumer wait.’
	control	dass Moritz sah, wie der Gärtner den Kunden warten ließ.
		that M.nom saw how the.nom gardener the.acc costumer wait let
		‘that Moritz saw how the gardener let the costumer wait.’
2 × 2	center	dass Moritz den Gärtner, der den Kunden warten ließ, arbeiten sah.
	embedded	that M.nom the.acc gardener who.nom the.acc costumer wait let work saw
		‘that Moritz saw the gardener that let the costumer wait work.’
	control	dass Moritz den Gärtner arbeiten sah, der den Kunden warten ließ.
		that M.nom the.acc gardener work saw who.nom the.acc costumer wait let
		‘that Moritz saw the gardener work that let the costumer wait.’
**CAUSATIVE VERB *LASSEN* TO LET—TRANSITIVE COMPLEMENT**		
1 × 3	center	dass Alexander den König den Dieb bestrafen lassen sah.
	embedded	that A.nom the.acc king the.acc thief punish let saw
		‘that Alexander saw the king letting punish the thief.’
	control	dass Alexander sah, wie der König den Dieb bestrafen ließ.
		that A.nom saw how the.nom king the.acc thief punish let
		‘that Alexander saw how the king let punish the thief.’
2 × 2	center	dass Alexander den König, der den Dieb bestrafen ließ, lachen sah.
	embedded	that A.nom the.acc king who.nom the.acc thief punish let laugh saw
		‘that Alexander saw the king that let punish the thief laugh.’
	control	dass Alexander den König lachen sah, der den Dieb bestrafen ließ.
		that A.nom the.acc king laugh saw who.nom the.acc thief punish let
		‘that Alexander saw the king laugh that let punish the thief.’
**CONTROL VERB *VERSUCHEN* TO TRY**		
1 × 3	center	dass Peter den Koch den Brand zu löschen versuchen sah.
	embedded	that P.nom the.acc cook the.acc fire to extinguish trying saw
		‘that Peter saw the cook trying to extinguish the fire.’
	control	dass Peter sah, wie der Koch den Brand zu löschen versuchte.
		that P.nom saw how the.nom cook the.acc fire to extinguish tried
		‘that Peter saw how the cook tried to extinguish the fire.’
2 × 2	center	dass Peter den Koch, der den Brand zu löschen versuchte, verzweifeln sah.
	embedded	that P.nom the.acc cook who.nom the.acc fire to extinguish tried despair saw
		‘that Peter saw the cook that tried to extinguish the fire despair.’
	control	dass Peter den Koch verzweifeln sah, der den Brand zu löschen versuchte.
		that P.nom the.acc cook despair saw who.nom the.acc fire to extinguish tried
		‘that Peter saw the cook despair that tried to extinguish the fire.’

*All sentences were introduced by the main clause “Ich weiSS” (“I know”)*.

The remaining eight sentences contained a verb cluster of the form “lexical verb – *versuchen* (“to try”) – *sah* (“saw”)”. In these sentences, the third NP was the object of the lexical verb, which always was a transitive verb, and the subject of the lexical verb was implicitly understood as the subject of the control verb *versuchen* (“to try”).

Sentences in the condition “center-embedded with 2×2 dependencies” were created from sentences in the condition “center-embedded with 1×3 dependencies” as follows. First, the *that*-clause now contained only the first two NPs, the subject and the first accusative NP. This NP was modified by a relative clause introduced by a subject relative pronoun. This relative clause contained the former second accusative NP. The relative clause ended in a 2-verb cluster containing the lexical verb and the second verb of the original 3-verb cluster. All *that*-clauses also ended in a 2-verb cluster with the verb *sah* (“saw”) as finite verb. The non-finite verb of the verb cluster was a lexical verb that did not occur in corresponding 1×3 sentences.

In this and the following two experiments, control sentences were derived from the experimental sentences by means of extraposition, thereby eliminating center embedding or at least reducing it. For the 1×3 sentences, control sentences were derived by extraposing the complement of the finite perception verb *sah* (“saw”), that is, the two infinitive verbs embedded below *saw* together with their arguments. Because a perception verb can take an infinitival complement only when this complement occurs to its left, the extraposed clause had to be turned into a finite clause introduced by *wie* (“how”). Despite this morpho-syntactic difference, the control sentences had the same meaning as the experimental sentences with center embedding. In the condition “2×2 dependencies,” the relative clause was extraposed behind the verb cluster of the *that*-clause. In this case, the extraposed clause had not to be modified in any way. Experimental and control sentences were thus identical with the exception of the position of the relative clause.

The 16 sentence quadruples were distributed onto four experimental lists according to a Latin square design. Each experimental list contained only one version of each sentence, with an equal number of sentences occurring in each of the four experimental conditions. Each experimental list was randomized and then combined with a list of 72 filler sentences. The filler sentences represented a variety of sentence structures and were partly taken from unrelated experiments.

#### 3.1.3. Procedure

Four written questionnaires were produced on the basis of the four lists of experimental and filler sentences. Participants completed the questionnaires as part of a class session. They were asked to judge the acceptability of each item on the questionnaire by marking one of the numbers 1 to 7 printed beneath each sentence. A scale ranging from 1 to 7 was chosen because such a scale is in common use (Schütze and Sprouse, 2014) and has proved its usefulness in numerous experiments (e.g., Weskott and Fanselow, 2011). A short instruction on the first page of the questionnaire told participants that 1 meant “totally unacceptable” and 7 meant “totally acceptable” (see the Supplementary Material for the complete instruction). The instruction did not contain any example sentences. Participants needed about 15–20 min to complete the questionnaire.

### 3.2. Results

All data presented in this paper were analyzed using the R statistics software, Version 3.3.2 (R Core Team, 2016). To test for significant effects, the judgment data were analyzed by means of mixed-effect modeling using the lme4 package (Bates et al., 2015b). The experimental factors and all interactions between them were entered as fixed effects into the model, using effect coding, that is, the intercept represents the unweighted grand mean and fixed effects compare factor levels to each other. In addition, the model included random effects for items and subjects with maximal random slopes supported by the data, following the strategy proposed in Bates et al. (2015a). The full model summary is reported as well as likelihood ratio tests, which assess the contribution of single factors or interactions. Where necessary, pairwise comparisons were computed using Tukey's test.

**Figure 1:**
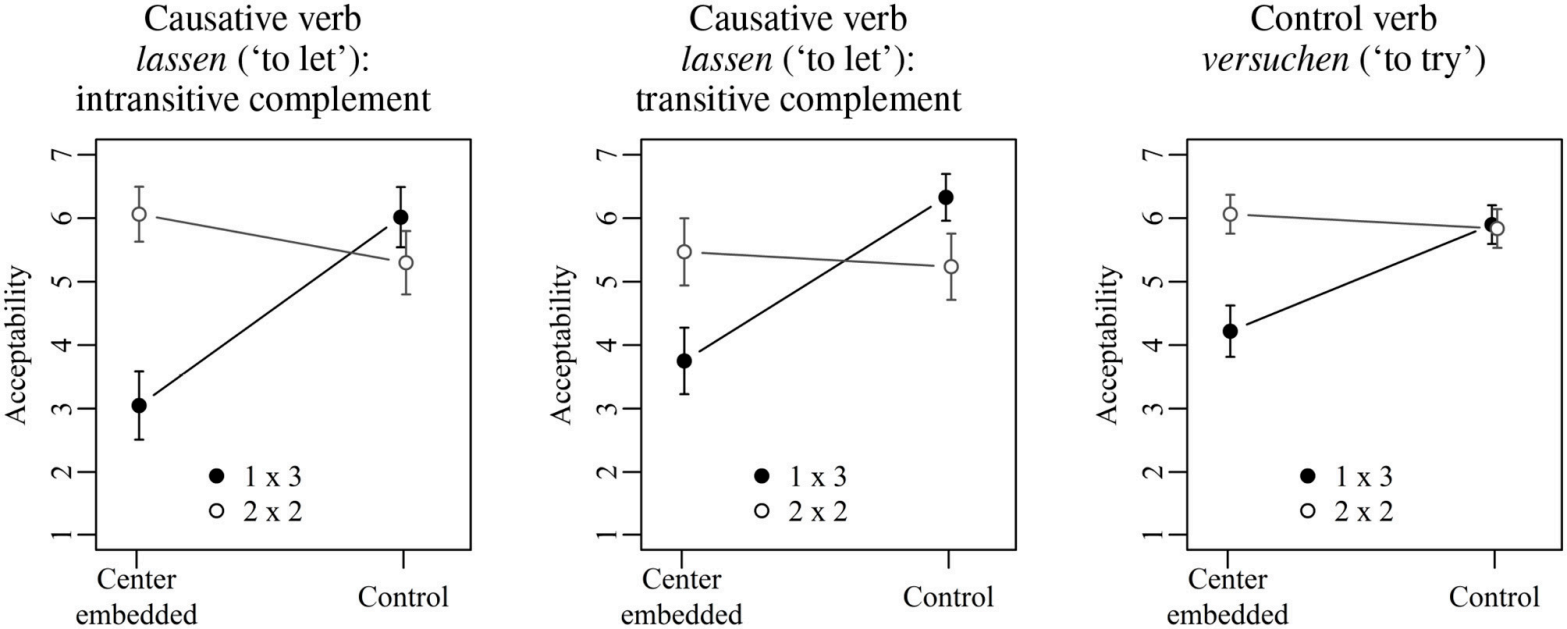
Mean acceptability ratings on a scale from 1 (low) to 7 (high) for Experiment 1. Error bars show 95% confidence intervals.

Figure 1 shows the mean acceptability ratings for the three sentence types investigated in Experiment 1. The basic pattern is the same in each case: 1×3 sentences with center embedding receive much lower mean ratings than 1×3 control sentences. In the 2×2 condition, in contrast, sentences with center embedding are judged as equally or even slightly more acceptable than control sentences. Although the exact mean ratings differ somewhat across the three sentence types, an initial statistical analysis including sentence type as a third factor showed neither a significant main effect of sentence type nor a significant interaction involving sentence type. The results thus do not depend on the specific combination of verbs with their associated lexical requirements, but on the more general structural configurations. The factor sentence type was accordingly dropped from the analysis.

**Figure 2:**
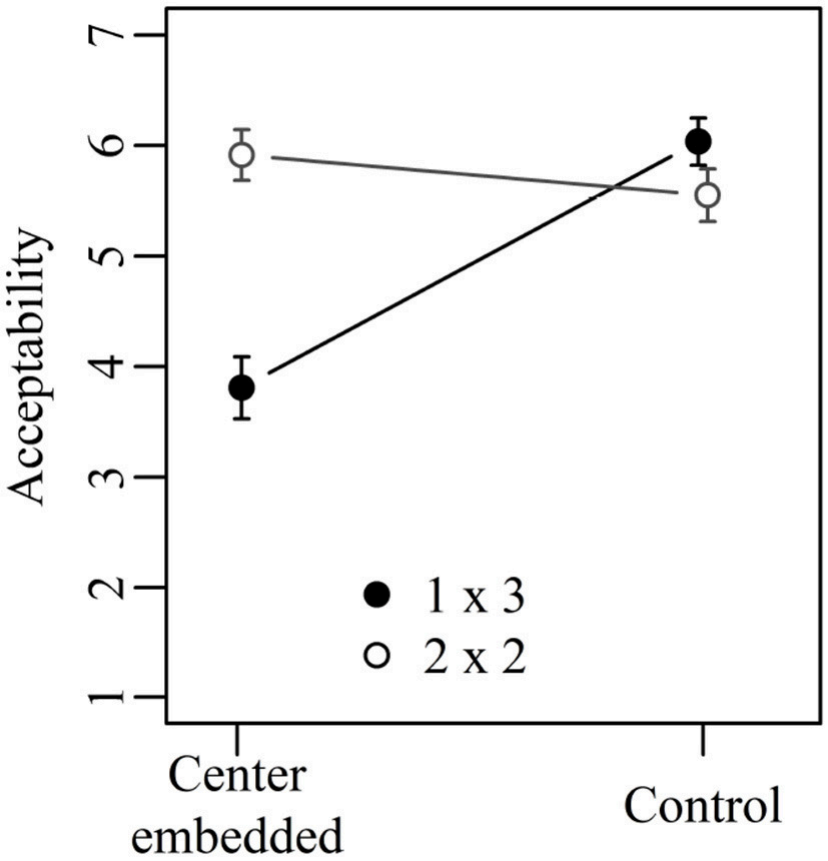
Mean acceptability ratings on a scale from 1 (low) to 7 (high) for Experiment 1. Error bars show 95% confidence intervals.

Figure 2 shows the mean acceptability ratings obtained in Experiment 1 collapsed across the three conditions of sentence type. The results of the corresponding statistical analysis are shown in Table 3. The two main effects as well as the interaction between them were significant. 1×3 sentences with center embedding received significantly lower acceptability ratings than 1×3 control sentences (3.8 vs. 6.0; Tukey's test: *t*-ratio = 10.21; *p* < 0.001). The acceptability of 2×2 sentences with center embedding, in contrast, did not differ significantly from 2×2 control sentences (5.9 vs. 5.6; Tukey's test: *t*-ratio = 1.67, *p* > 0.1). Furthermore, there was no significant acceptability difference when comparing 1×3 control sentences and 2×2 control sentences (6.0 vs. 5.6; Tukey's test: *t*-ratio = 2.05, *p* > 0.1), whereas for center-embedded sentences, the corresponding comparison was significant (3.8 vs. 5.9; Tukey's test: *t*-ratio = 8.90; *p* < 0.001).

**Table 3:** Linear mixed model fitted by maximum likelihood estimation for Experiment 1, including p-values from likelihood ratio tests.

	**Coefficient**	**Std. Error**	***t*-value**	***p* (LRT)**
(Intercept)	5.3271	0.1696	31.412	
Structure	−0.9316	0.1413	−6.592	<0.001
Dependencies	−0.8105	0.1684	−4.814	<0.001
Structure:dependencies	−2.5898	0.3322	−7.795	<0.001

Acceptability ~ structure ^*^ dependencies + (dependencies || subject) + (structure ^*^ dependencies || sentence)

### 3.3. Discussion

Experiment 1 yielded two major results. First, sentences with three nested dependencies all originating in a single 3-verb cluster are difficult to process. This replicates the original finding of Bach et al. (1986). The new finding of Experiment 1 is that 2×2 sentences are more acceptable than 1×3 sentences, and in fact no less acceptable than control sentences containing the same number of dependencies but with a maximum number of 2 nestings. This contradicts De Vries et al.'s (2011) generalization that sentences containing three or more nested dependencies pose special challenges to the human parser. Thus, the Incomplete Dependency Hypothesis in (3) is incorrect even if restricted to nested dependencies, which are all distinct in the sense of connecting each argument to a separate head.

Given the results of Experiment 1, the difficulty of the 3-verb clusters considered here cannot be attributed to general limitations of the human parsing mechanism with regard to the processing of nested dependencies. This leaves us with the question of why verb clusters with more than two verbs lead to heavy processing load in cases where each verb introduces arguments of its own. The Verb-Cluster Complexity Hypothesis in (11) provides an answer to this question, but it is specifically tailored to the case of verb-cluster formation. It should therefore be accepted only if the findings of Experiment 1 cannot be accounted for by general theories of syntactic complexity.

In section 2, two general sources of sentence complexity were discussed in addition to the number of open nested dependencies, namely integration cost capturing dependency distance and storage cost capturing phrase-structure complexity. As shown in detail in the Supplementary Material, integration and storage cost do not provide an account for the low acceptability of Dutch and German verb clusters with 3 or more argument-taking verbs. This does not argue against the explanatory potential of these notions, but instead points to the conclusion that verb-cluster formation by itself can result in enhanced processing complexity under certain circumstances. In order to unterstand what makes verb clusters hard to process, the empirical findings concerning the processing complexity of sentences with verb clusters are summarized below.

- Verb clusters with only one argument-taking verb are (relatively) easy even if containing up to 5 verbs (Bader and Schmid, 2009b; Bader et al., 2009).
- Verb clusters with two argument-taking verbs are easy (Bach et al., 1986, Experiments 1 and 2; see also Nakatani, 2006, for related evidence from Japanese).
- Verb clusters with more than two argument-takings verbs are difficult (Bach et al., 1986, Experiment 1).
- Passivization of the argument structure resulting from combining two argument-taking verbs is difficult (Bader and Schmid, 2009a).

Based on these findings, (15) gives a descriptively more adequate formulation of the Verb-Cluster Complexity Hypothesis of Bader and Schmid (2009a).

(15) *Verb-Cluster Complexity Hypothesis (revised)*
Operating on a composite argument structure derived by
  verb-cluster formation is costly for the human parser.

Combining two argument-taking verbs creates a composite argument structure. This is an easy task for the parser, but applying further operations to such a composite argument structure is difficult. These further operations can be of two types. First, a third argument-taking verb is added, as in the sentences of Bach et al. (1986) and Experiment 1. Second, an argument-structure changing auxiliary is added, as in the long passive construction investigated by Bader and Schmid (2009a). Adding a verb that has no effect on the argument structure of the verb cluster it combines with (e.g., a perfect auxiliary) is easy. In sum, working on simple argument structures as they are associated with verbs is easy for the parser, but working on composite argument structures is difficult. A possible reason for this could be that composite argument structures cannot be retrieved from the lexicon but must be computed on the fly. The need to hold the resulting complex argument structure in working memory and simultaneously to work on it might be the source of the observed difficulty.

A final issue concerning verb-cluster formation is why Bach et al. (1986) found Dutch crossed dependencies to be somewhat more acceptable than German nested dependencies. As noted above, the size of this effect was rather small, and several minor advantages brought about by the Dutch order could be responsible for it. First, the order of verbs in Dutch is better suited for incremental parsing and interpretation than the order of verbs in German. Consider first Dutch. The crossed dependencies of Dutch are a consequence of the fact that the hierarchically highest verb V_1_ comes first, followed by V_2_, that is, the verb selected by V_1_, and so on. Verbs thus appear in the same order as in English. Due to this ordering, Dutch verb clusters can be syntactically analyzed and semantically interpreted incrementally as each verb is encountered. The first verb to be encountered is the finite verb. This verb can be linked to the first NP, the subject NP, and a preliminary semantic analysis can be computed with an open slot for the missing verbal complement. This open slot can be filled on encountering the second verb and the second NP can be linked. There will now be an open slot for the verbal complement of the second verb, which is filled as soon as the third verb is encountered.

Since verbs in German appear in reversed order, parsing and interpretation cannot be fully incremental. When the first verb of the cluster is encountered, the third NP can be linked as its subject argument, but how the verb is related to the already build syntactic structure or to the partial semantic representation computed so far cannot be determined, because this verb is a non-finite verb, but a finite verb is required to make contact with the existing higher level structures. The second verb is again a non-finite verb, so making the connection with the higher level structure has still to wait, although linking of its subject argument is possible. Only when the third verb, the finite verb, is encountered, is it possible to fully integrate the syntactic structure and the semantic representation of the embedded clause into those of the matrix clause.

The processing advantage for crossed dependencies with regard to incremental parsing does not seem to be a large one, but the acceptability difference found by Bach et al. (1986) was not large either. Furthermore, other factors may also contribute to this difference. For example, it has been hypothesized that the order of the arguments associated with a verb reflect their hierarchical position within the semantic representation of the verb (e.g., Bierwisch, 1986). The agent is the highest argument in the semantic representation (as the first argument of the causal relation), and at the same time the argument that precedes all other arguments. Given this hypothesis, a Dutch verb cluster, where the semantically highest verb comes first, is advantageous because the order of verbs parallels the order of arguments.

In the remainder of this paper, sentences with four nested dependencies and verb clusters containing at most two verbs will be explored more closely. Experiment 2 investigates the complexity of sentences for which storage cost and integration cost make opposite predictions. The final Experiment 3 takes a closer look at integration cost in sentences matched for storage cost.

## 4. Experiment 2

As noted above, prior research on sentence complexity in verb-final languages has revealed an anti-locality effect: additional material in front of the clause-final verb leads to shorter reading times on the verb (e.g., Konieczny, 2000; Vasishth and Lewis, 2006; Nakatani and Gibson, 2008; Levy and Keller, 2013). Locality effects have also been found, however. Levy and Keller (2013) investigated sentences as in (16), varying whether or not the relative clause contained the adverbial and the dative object.

(16) Der Mitschüler, der          (zur Ahndung) (dem
       the  classmate,  who.nom as    payback   the.dat
       Sohn) den        Fußball versteckt  hat, …
       son     the.acc football hidden     has
       ‘The classmate who hid the football from the son as
       payback …’

Reading times on the relative clause verb were shorter when the dative object was included but longer when the adverbial phrase was included. Following Konieczny (2000), Levy and Keller (2013) explain this in terms of expectations. When the relative clause contains a dative object in addition to an accusative object, a more specific prediction concerning the upcoming verb is possible, making the integration of the verb easier. An additional adverbial phrase, in contrast, is of no help in predicting the verb. In this case, reading times go up due to the lengthened dependency. Thus, both integration cost and verb-specific expectations seem to affect processing cost in verb-final clauses.

What has not been investigated so far is how integration cost and storage cost jointly affect the acceptability of sentences with multiple center embedding. In contrast to the verb specific expectations manipulated in investigations of the anti-locality effect, storage cost is a measure of the number of expectations that the parser has to retain at each point during the parse. Experiment 2 investigates sentences containing four nested dependencies for which integration cost and storage cost make opposing predictions. One type of sentences is similar to the sentences in the 2×2 condition of Experiment 1. This sentence type is illustrated in (17).

(17) Ich weiß, dass Peter         die        Behauptung, dass
       I    know that  Peter.nom the.acc claim           that
       der          Moderator den        Sänger auftreten ließ und
       the.nom host          the.acc singer  perform  let   and
       dann kündigte, zu entkräften   versuchte.
       then  resigned  to  refute         tried
       ‘I know that Peter tried to refute the claim that the host
       let the singer perform and then resigned’

Like the 2×2 sentences of Experiment 1, sentence (17) contains one degree of center embedding. As shown in Table 4, both the matrix clause and the embedded clause contain a 2-verb cluster and thus four nested dependencies, two within the matrix clause and two within the embedded clause. While sentences as in (17) are similar to the 2×2 sentences of Experiment 1 with regard to their basic structure (4 nested dependencies distributed across two separate 2-verb clusters), there are also two differences. First, the center-embedded clause in (17) is not a relative clause but a complement clause. This change was made because complement clauses do not involve traces. Traces are a controversial issue in syntactic theory and theories of the human parser alike. By investigating complement clauses instead of relative clauses, these controversies are circumvented when integration cost profiles are computed. The second difference is that the 2-verb cluster in the center-embedded clause is followed by a conjoined VP. This conjoined VP does not increase the degree of nesting but increases the distance between the verb cluster of the upper *that*-clause and its arguments.

**Table 4:**
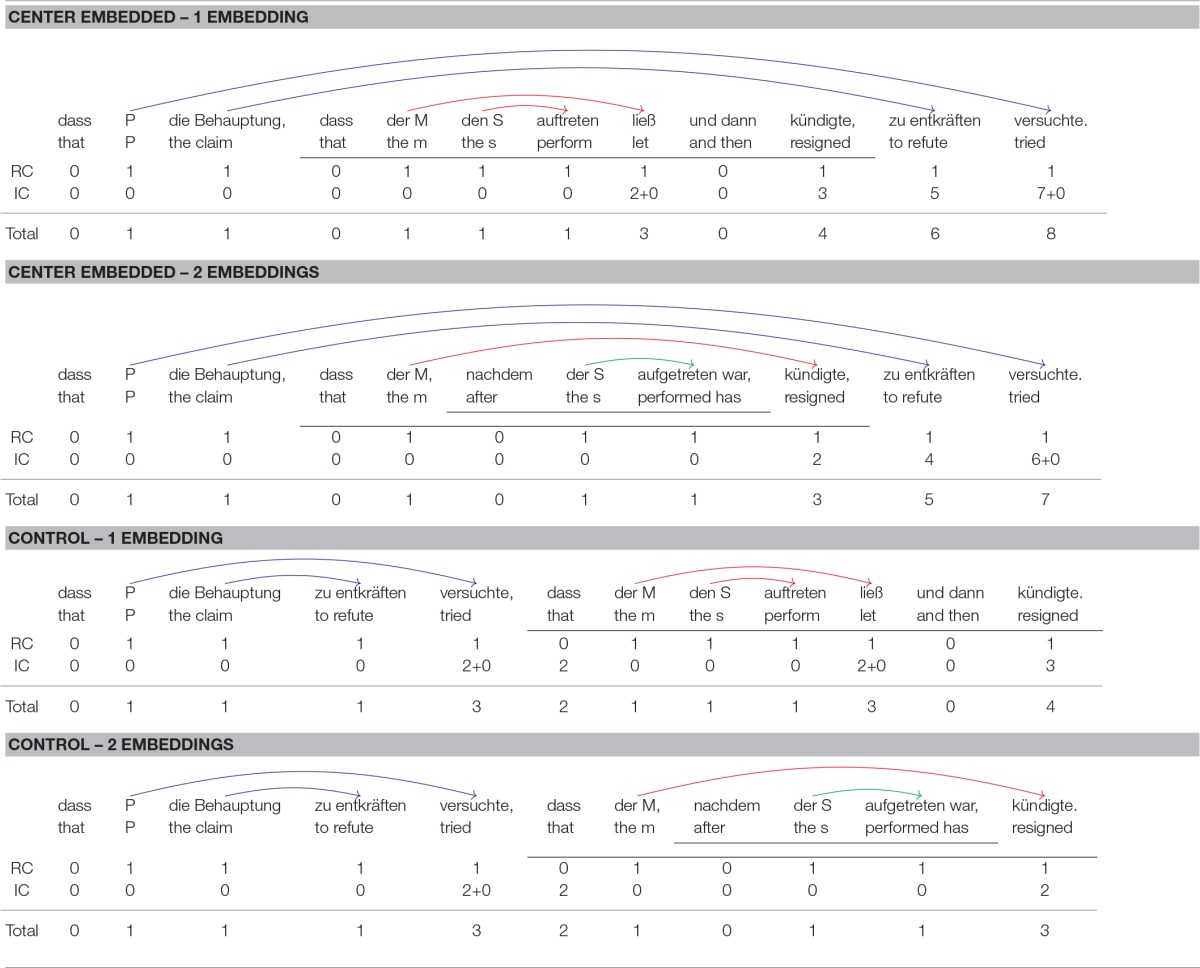
Syntactic dependencies and integration cost profiles for the sentence conditions of Experiment 2.

*RC, referential processing cost; IC, structural integration cost; total, total processing cost*.

Sentences as in (17) will be compared to sentences as in (18).

(18) Ich weiß, dass Peter          die        Behauptung, dass
       I    know that  Peter.nom  the.acc claim           that
       der          Moderator, nachdem der         Sänger
       the.nom host            after        the.nom singer
       aufgetreten war, kündigte, zu entkräften versuchte.
       performed  has  resigned  to  refute       tried
       ‘I know that Peter tried to refute the claim that the host
       resigned after the singer had performed.’

As shown in Table 4, sentence (18) again contains four nested dependencies, but this time distributed across three verb clusters. The upper *that*-clause contains a 2-verb cluster. Center-embedded within the upper *that*-clause is a second *that*-clause which contains a 1-verb cluster. The lower *that*-clause in turn hosts a center-embedded temporal clause which also contains a 1-verb cluster. The degree of center embedding is two in sentence (18).

Storage cost is greater in (18) than in (17), but the reverse is true for integration cost. Consider first storage cost. When processing the most deeply embedded clause in sentence (17), the parser must keep two predicted verbal heads in memory—one verb for each *that*-clause. For sentence (17), one additional predicted verb must be kept in memory, the verb of the temporal clause embedded within the lower *that*-clause. Thus, a maximal storage cost of two for sentence (17) contrasts with a maximal storage cost of three for sentence (18). According to the Predicted Syntactic Head Hypothesis in (6), sentence (18) should therefore be more difficult to process than sentence (17).

Consider next integration cost, which is also shown in Table 4. The first line below each sentence gives the referential processing cost (RC), the second line the structural integration cost (IC), and the final line the total processing cost, which is the sum of referential and integration cost. Consider first the integration cost profile for center embedded sentences with one level of embedding. Each NP and each verb introduces a new discourse referent and is therefore associated with a referential cost of 1. As for structural integration cost, the following considerations apply:

- *Paul*, *die Behauptung* (“the claim”), *der Moderator* (“the host”), *den Sänger* (“the singer”): There is no head with which the NPs could integrate directly after they have been encountered in the input. Structural integration cost is therefore 0 for all of them.
- *auftreten* (“perform”): The first verb that the parser encounters in the input can be integrated with the final NP *den Sänger*, which fills the subject argument role of *auftreten*. Integration cost is zero because the two items occur adjacent to each other.
- *ließ* (“let”): Integrating *ließ* with its subject *der Moderator* (“the host”) spans two new discourse referents, *den Sänger* (“the singer”) and *auftreten* (“perform”). This verb must also integrate with its verbal complement *auftreten*, but because the two verbs occur adjacent to each other, no structural integration costs ensue.
- *kündigte* (“resigned”): This verb also integrates with the NP *der Moderator* (“the host”). Three new discourse referents intervene between the verb and its subject.
- *zu entkräften* (“refute”): The integration of this verb with its object *die Behauptung* (“the claim”) spans 5 new discourse referents.
- *versuchte* (“tried”): This verb must integrate with its subject and with its verbal complement. The former integration spans 7 new discourse referents whereas the latter spans zero new discourse referents.

Center embedded sentences with two levels of embedding have the same integration cost profile up to and including the first verb in the left-to-right parse. Because the verb *ließ* (“let”) does not occur in 2-embedding sentences, the final three verbs are associated with a smaller integration cost in 2-embedding sentences than in 1-embedding sentences. For example, the verb *kündigte* (“resigned”) is now the second verb. As before, it must integrate with the NP *der Moderator* (“the host”). This integration spans only two new referents (singer, perform) in contrast to three for 1-embedding sentences (singer, perform, let). For the last two verbs, integration cost is similarly diminished by one unit in 2-embedding sentences.

For center embedded sentences with one level of embedding, the maximum integration cost is 8 whereas for center embedded sentences with two levels of embedding the maximum integration is only 7. If we assume with Gibson (2000) that the acceptability ratings for a sentence reflect its maximum integration cost, we get the prediction that center embedded sentences with two levels of embedding should be more acceptable than center embedded sentences with one level of embedding. The same holds for summed integration cost, which is obtained by summing up the integration cost for each word. Summed integration cost is 26 for center embedded sentences with one level of embedding but only 20 for center embedded sentences with two levels of embedding.

As in Experiment 1, control sentences in Experiment 2 were derived from experimental sentences by means of extraposition. As shown in Table 4, the most deeply embedded *that*-clause was put behind the higher *that*-clause. For 1-embedding sentences, this removes center embedding completely, and at no point during the ongoing parse the parser has to keep more than a single predicted verb in memory. For 2-embedding sentences, the extraposed *that*-clause still contains a center-embedded temporal clause. When processing this temporal clause, two predicted verbs must be kept in memory. The maximum storage cost for control sentences is therefore lower than for center-embedded sentences, but the difference between the two types of control sentences is the same as the difference between the two center-embedded sentences (2 vs. 1 for control sentences, 3 vs. 2 for center-embedded sentences). Integration cost is similarly reduced in control sentences in comparison to center-embedded sentences. Furthermore, the control sentences are similar to the center embedded sentences in that integration cost is lower in sentences with two embeddings than in sentences with one embedding. This holds for maximum and summed integration cost alike.

In sum, integration cost is higher in 1-embedding sentences than in 2-embedding sentences, but storage cost is higher in 2-embedding sentences than in 1-embedding sentences. This holds for center embedded and for control sentences alike, although storage and integration cost are lower in the latter than in the former.

### 4.1. Methods

#### 4.1.1. Participants

Fourty students from the Goethe-University Frankfurt participated in Experiment 2. All participants were native speakers of German and naive with respect to the purpose of the experiment. Ethical approval was not required for this study in accordance with the national and institutional guidelines.

#### 4.1.2. Materials

Sixteen sentences were constructed for Experiment 2, with each sentence appearing in four versions according to the two factors Embedding (1 vs. 2) and Structure (center embedded vs. control). Most sentences were based on the lexical material of the sentences investigated in Experiment 1. The two verbs *versuchen* (“to try”) and *lassen* (“to let”) were used again as verbs selecting a verbal complement. In order to test whether the acceptability of the sentences under consideration is mainly governed by structural factors, not by lexical factors, the position of *versuchen* (“to try”) and *lassen* (“to let”) was varied as a within-item factor.

All sentences again started with the main clause “Ich weiß” (“I know”), followed by a *that*-clause. All *that*-clauses consisted of a proper name as subject, a definite NP as accusative object and a 2-verb cluster. Eight sentences contained a verb cluster with a non-finite lexical verb and the finite verb *ließ* (“let”). The verb cluster of the other eight sentences contained a non-finite lexical verb and the finite control verb *versuchte* (“tried”). Table 5 shows an example of each sentence type.

**Table 5:** Two stimulus sentences from Experiment 2, one with a causative verb in the most deeply embedded clause and a control verb in the dominating matrix clause and one with the reversed positions of causative and control verb.

**CONTROL VERB IN HIGHER CLAUSE, CAUSATIVE VERB IN LOWER CLAUSE**		
1 embedding	Center	dass Moritz das Gerücht, dass der Architekt den Gärtner
	embedded	that M.nom the.acc rumor that the.nom architect the.acc gardener
		warten ließ und stattdessen frühstückte, zu verbreiten versuchte.
		wait let and instead had breakfast to disseminate tried
		‘that Moritz tried to disseminate the rumor that the architect let the gardener wait and had breakfast instead.’
	Control	dass Moritz das Gerücht zu verbreiten versuchte, dass der Architekt
		that M.nom the.acc rumor to disseminate tried that the.nom architect
		den Gärtner warten ließ und stattdessen frühstückte.
		the.acc gardener wait let and instead had breakfast
2 embeddings	Center	dass Moritz das Gerücht, dass der Architekt, während der Gärtner
	embedded	that M.nom the.acc rumor that the.nom architect while the.nom gardener
		gewartet hat, frühstückte, zu verbreiten versuchte.
		waited has had breakfast to disseminate tried
		‘that Moritz tried to disseminate the rumor that the architect had breakfast while the gardener waited.’
	Control	dass Moritz das Gerücht zu verbreiten versuchte,
		that M.nom the.acc rumor to disseminate tried
		dass der Architekt, während der Gärtner gewartet hat, frühstückte.
		that the.nom architect while the.nom gardener waited had had breakfast
**CAUSATIVE VERB IN HIGHER CLAUSE, CONTROL VERB IN LOWER CLAUSE**		
1 embeddings	Center	dass der Wirt die Behauptung, dass der Koch den Brand
	embedded	that the.nom landlord the.acc claim that the.nom cook the.acc fire
		zu löschen versuchte und dann verzweifelte, verbieten ließ.
		to extinguish tried and then despaired banned got
		‘that the landlord let ban the claim that the cook tried to extinguish the fire and despaired.’
	Control	dass der Wirt die Behauptung verbieten ließ,
		that the.nom landlord the.acc claim banned got
		dass der.nom Koch den.acc Brand zu löschen versuchte und dann verzweifelte.
		that the cook the fire to extinguish tried and then despaired
2 embeddings	Center	dass der Wirt die Behauptung, dass der Koch,
	embedded	that the.nom landlord the.acc claim that the.nom cook
		nachdem der Brand gelöscht war, verzweifelte, verbieten ließ.
		after the.nom fire extinguished was despaired banned got
		‘that the landlord let ban the claim that the cook despaired after the fire had been extinguished.’
	Control	dass der Wirt die Behauptung verbieten ließ,
		that the.nom landlord the.acc claim banned got
		dass der Koch, nachdem der Brand gelöscht war, verzweifelte.
		that the.nom cook after the.nom fire extinguished was despaired

*All sentences were introduced by the main clause “Ich weiß” (“I know”). Center-embedded and control sentences have the same meaning and only differ with regard to the position of the embedded clauses. A translation is therefore only given for the center-embedded condition*.

The accusative object in all *that*-clauses was a definite NP with a head noun selecting a *that*-clause itself. In 1-embedding sentences, this second *that*-clause started with the subject, followed by an accusative object and a 2-verb cluster. This cluster consisted of a non-finite lexical verb and either the finite verb *ließ* (“let”) or the finite verb *versuchte* (“tried”). When *ließ* appeared in the inner *that*-clause, *versuchte* appeared in the outer *that*-clause, and vice versa. The 2-verb cluster of the inner *that*-clause was followed by the conjunction und (“and”), a one-word adverbial and a finite lexical verb. For control sentences, the lower *that*-clause was extraposed behind the upper *that*-clause.

2-embedding sentences differed from 1-embedding sentences as follows. The lower *that*-clause now consisted only of the former subject and the lexical verb that follows the conjunction in 1-embedding sentences. The accusative object and the lexical verb of the 2-verb cluster in 1-embedding sentences were used to construct an adverbial clause that was center-embedded within the lower *that*-clause. The former accusative object was always the subject in this adverbial clause. Control sentences were again created by extraposing the lower *that*-clause behind the higher *that*-clause.

#### 4.1.3. Procedure

As in Experiment 1, participants received a written questionnaire and had to rate the acceptability of each sentence on a scale from 1 (“totally unacceptable”) to 7 (“totally acceptable”).

### 4.2. Results

The statistical analysis proceeded as for Experiment 1. An initial inspection revealed that the order of the two verbs *lassen* (“let”) and *versuchen* (“try”) (see Table 5) had no effect on acceptability. In all four combinations of the two factors Embedding and Structure, the difference between the two verb orders was less than 0.3, and verb order as a third factor within the statistical model was not involved in any significant effects. The results of Experiment 1 thus seem to reflect the particular syntactic configurations under investigation and not verb-specific idiosyncrasies. The factor verb order was accordingly dropped from all further analyses.

**Figure 3:**
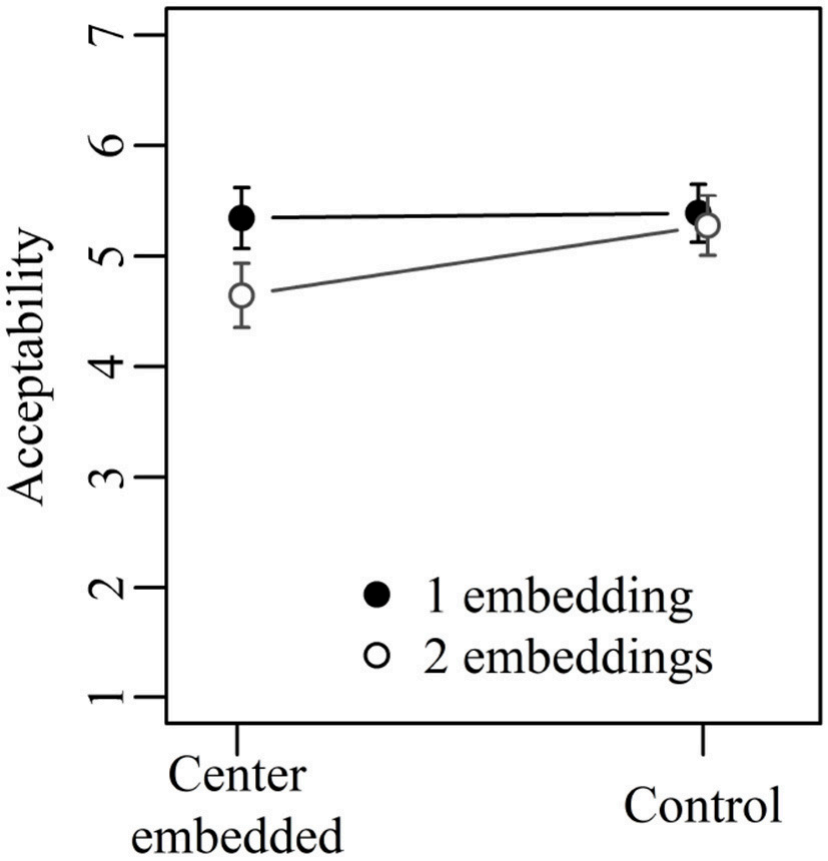
Mean acceptability ratings on a scale from 1 (low) to 7 (high) for Experiment 2. Error bars show 95% confidence intervals.

Figure 3 shows the mean acceptability ratings for Experiment 2 collapsed across verb order. The results of the corresponding mixed-effect model are given in Table 6. Both the two main effects and the interaction between them were significant. Center embedded sentences with one embedding did not differ significantly from corresponding control sentences (5.3 vs. 5.4; Tukey's test: *t*-ratio = 0.22; n.s.). Center-embedded sentences with two embeddings, in contrast, were judged as significantly less acceptable than corresponding control sentences (4.6 vs. 5.3; Tukey's test: *t*-ratio = 3.17; *p* < 0.05). The two types of control sentences did not differ significantly from each other (5.4 vs. 5.3; Tukey's test: *t*-ratio = 0.58; n.s.) but the two types of center embedded sentences did (4.6 vs. 5.3; Tukey's test: *t*-ratio = 3.63; *p* < 0.01).

**Table 6:** Linear mixed model fitted by maximum likelihood estimation for Experiment 2, including p-values from likelihood ratio tests.

	**Coefficient**	**Std. Error**	***t*-value**	***p* (LRT)**
(Intercept)	5.1625	0.1859	27.769	
Structure	−0.3375	0.1525	−2.213	<0.01
Embeddings	0.4063	0.1435	2.832	<0.01
Structure:embeddings	0.5875	0.2570	2.286	<0.05

Acceptability ~ structure ^*^ embedding + (structure + embedding || subject) + (structure ^*^ embedding || sentence).

### 4.3. Discussion

Experiment 2 investigated two types of center-embedded sentences that were matched in terms of the number of nested dependencies—they contained always four nested dependencies—but differed in terms of storage and integration cost. Structural integration cost was greater in sentences with one embedding than in sentences with two embeddings, whereas storage cost was greater in 2-embedding sentences than in 1-embedding sentences. Since center-embedded sentences with one embedding were judged as more acceptable than center-embedded sentences with two embeddings, Experiment 2 allows the conclusion that storage cost (as measured by the number of predicted heads) is more important than integration cost (as measured by dependency length). In addition, Experiment 2 strengthens the conclusion reached in Experiment 1 that the number of nested dependencies is not a good predictor for sentence complexity. Despite containing four nested dependencies, center-embedded sentences with one embedding were as acceptable as their control sentences, which at no point contained more than two nested dependencies.

The current results show that sentence complexity increases with the number of verbs that have to be predicted. This contrasts with cases where predictions get more specific due to the presence of more arguments, as in the sentences exhibiting the anti-locality effect. For them, more specific predictions decrease complexity according to the most common interpretation of the anti-locality effect (Konieczny, 2000; Vasishth and Lewis, 2006; Levy and Keller, 2013). Taken together, this suggests that predictions help the parser unless too many predictions have to be made simultaneously.

An additional finding of Experiment 2 was that control sentences for both 1- and 2- embedding sentences were judged as equally acceptable, despite showing the same difference in terms of storage cost as the center-embedded sentences. In embedding 1 control sentences, the maximal number of predicted verbs was one whereas it was two in embedding 2 control sentences. Taken together with the results for the center-embedded sentences, we thus see a decrease in acceptability when the number of predicted heads increases from two to three, but not when it increases from one to two.

## 5. Experiment 3

Four nested dependencies can be realized by three combinations of verbs and verb clusters: two 2-verb clusters, a 2-verb cluster and two single verbs, and four single verbs. The first two configurations were investigated in Experiments 1 and 2. The third experiment investigates the last configuration—each dependency originates in a verb of its own. An example sentence is given in (19). Because there is a verb for each dependency and dependencies are nested, sentence (19) contains three levels of center embedding.

(19) Der        Vorwurf, dass mein       Kollege    jeden
       the.nom charge     that  my.nom colleague every.acc
       song  den         ein      Sänger, den         der          Chef
       Song, that.acc a.nom singer   that.acc  the.nom  boss
       nicht kennt, singt, ablehnt, stimmt.
       not   knows sings  rejects   is-right
       ‘The charge that my colleague rejects every song that a
       singer that the boss does not know sings, is true.’

The first aim of Experiment 3 was to test whether sentences with three levels of center embedding lead to clear unacceptability or whether acceptability degrades in a more gradient way. The second aim of Experiment 3 was to test whether integration cost affects the acceptability of sentences that are matched in terms of storage cost. Integration cost is manipulated by varying the number of new discourse referents spanned by the dependencies in complex sentences as in (19). Like all the sentences investigated so far, all NPs in sentence (19) are full NPs with the exception of the relative pronouns. According to the DLT, each full NP introduces a new discourse referent. This distinguishes full NPs from pronominal NPs, which do not introduce new discourse referents. They are therefore not associated with a referential processing cost and they do not count for the computation of structural integration cost. Evidence for this assumption has been provided by Warren and Gibson (2002), who have shown that English doubly center-embedded relative clauses are easier to comprehend when the most deeply embedded relative clause contains a pronoun instead of a full NP.

Experiment 3 compares sentences like (19) to sentences like (20). Here, two of the full NPs of sentence (19) have been replaced by first-person pronouns. Two NPs were replaced by pronouns in order to increase the chance of observing an effect of integration cost in case such an effect exists.

(20) Der       Vorwurf, dass ich       jeden       Song,
       the.nom charge    that  i.nom every.acc song
       den        ein      Sänger, den         ich      nicht kenne,
       that.acc a.nom singer  that.acc i.nom not    know
       singt, ablehne, stimmt.
       sings  reject     is-right
       ‘The charge that I reject every song that a singer that I do
        not know sings, is true.’

Table 7 shows the integration cost profiles for the sentences investigated in Experiment 3. For each verb, integration cost is higher in the high-load condition, which contains full NPs throughout, than in the low-load condition, in which two full NPs have been replaced by a pronoun. The first verb (*kenne* “know”), for example, must integrate with its subject and its object. The subject is adjacent to the verb and therefore no structural integration cost ensues. The object, that is, the relative pronoun, is separated from the verb by the subject. When the subject is a full NP, structural integration cost is one, but when the subject is a pronoun, structural integration cost is again zero. Similar considerations apply to the remaining verbs. For them, the difference between the high- and low-load condition is always two, either because the verb must integrate with two arguments (*singt* “sings,” *ablehnt* “rejects”) or because two pronouns intervene (*stimmt* “is correct”). Integration cost is highest on the penultimate verb (*ablehnt* “rejects”). In sum, the maximum integration cost is 10 in the high-load condition and 8 in the low-load condition. Summed integration cost, which is obtained by summing up the integration cost for each word, is 31 in the high-load condition and 22 in the low-load condition. Sentences in the low-load condition should therefore be rated as more acceptable than sentences in the high-load condition.

**Table 7:**
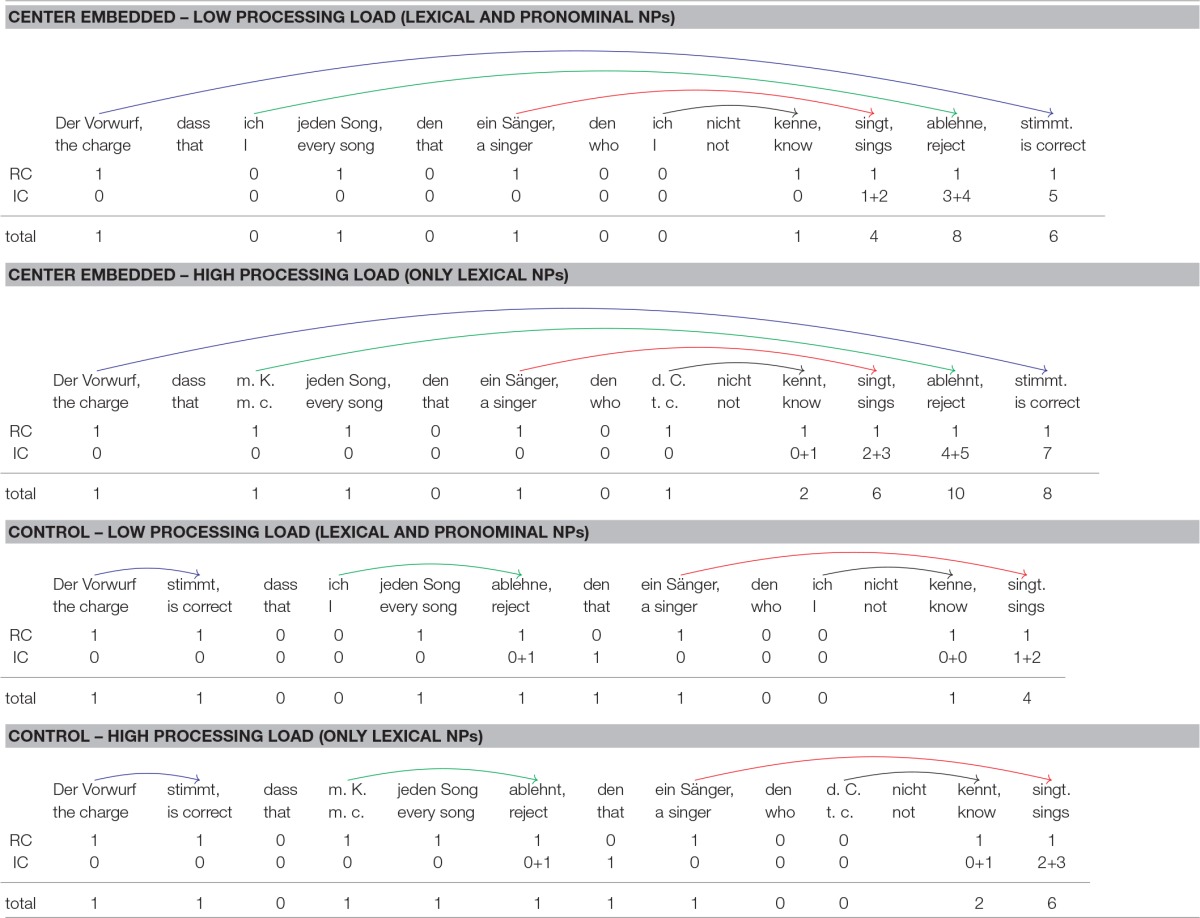
Syntactic dependencies and integration cost profiles for the sentence conditions of Experiment 3.

*RC, referential processing cost; IC, structural integration cost; total, total processing cost*.

Extraposition was again used for deriving control sentences from center-embedded sentences, as also shown in Table 7. Because of the high degree of center embedding, extraposition was applied twice for deriving control sentences. This removes center embedding with the exception of the most deeply embedded relative clause, which is still center-embedded in the control sentences. The maximum integration cost for control sentences is 6 in the high-load condition but only 4 in the low-load condition. Thus, maximal integration cost is lower in control than in center-embedded sentences, but the difference between high- and low-load is identical for experimental and for control sentences. Summed integration cost in control sentences is 15 in the high-load and 11 in the low-load condition and thus lower than in center-embedded sentences. In sum, high-load sentences should be less acceptable than low-load sentences, and center-embedded sentences should be less acceptable than control sentences. This prediction holds for maximum as well as summed integration cost.

In contrast to the main effects of load and structure, the predictions for the interaction between the two factors differ between maximum and summed integration cost. As shown above, for maximum integration cost the difference between low- and high-load condition is 2 for both center-embedded and control sentences. For summed integration cost, the difference between high- and low-load condition is 15 − 11 = 4 for the control sentences but 31 − 22 = 9 for the center-embedded sentences. Summed integration cost therefore predicts an interaction between load and structure whereas maximum integration cost predicts additive effects. By looking at the interaction, we can thus test the hypothesis of Gibson (2000) that acceptability reflects maximum integration cost and not summed integration cost. This assumption could not be tested in Experiment 2 because there any potential effect of integration cost was offset by an opposite effect of storage cost.

### 5.1. Methods

#### 5.1.1. Participants

Fourty students from the Goethe-University Frankfurt participated in Experiment 3. All participants were native speakers of German and naive with respect to the purpose of the experiment. Ethical approval was not required for this study in accordance with the national and institutional guidelines.

#### 5.1.2. Materials

For Experiment 3, sixteen new sentences were constructed, with each sentence appearing in four versions according to the two factors Load (low vs. high) and Structure (center embedded vs. control). A sentence in all of its four versions is shown in Table 8. Each sentence started with a noun phrase that was the subject of the main clause. The remainder of the main clause made a predication about the subject NP. The head noun of the subject was always a noun taking a sentential complement in the form of a *that*-clause. This clause appeared either adjacent to the head noun (condition center-embedded) or after the main clause (condition control). The *that*-clause consisted of a subject, an accusative object and a verb. The subject was either the first-person pronoun *ich* (“I”) (condition low load) or a full lexical NP (condition high load). The object of the *that*-clause was modified by a relative clause that either appeared directly behind the object (condition center-embedded) or behind the *that*-clause (condition control). In half of the sentences, this relative clause consisted of a subject relative pronoun, a *von* (“by”) PP, and a verb in the passive voice; in the other half of the sentences, the relative clause consisted of an accusative relative pronoun, a subject and an active verb. The second NP in each relative clause was modified by a second relative clause that always appeared adjacent to its head noun. This relative clause was introduced by an accusative relative pronoun in eight sentences and by a relative pronoun contained within a PP in the other eight sentences. The subject of the relative pronoun was either the first-person pronoun *ich* (“I”) (condition low load) or a full lexical NP (condition high load).

**Table 8:** A complete stimulus sentence from Experiment 3.

**LOW PROCESSING LOAD**		
Center	Das Gerücht, dass ich jedes Rezept, das ein Koch, den ich
embedded	the.nom rumor that I.nom every.acc recipe that.acc a.nom chef who.acc I.nom
	aus dem Fernsehen kenne, kreiert, nachkoche, ist frei erfunden.
	from TV know creates cook is freely fictitious
	‘The rumor that I cook every recipe that a cook that I know from TV creates is a complete fabrication.’
Control	Das Gerücht ist frei erfunden, dass ich jedes Rezept nachkoche,
	the.nom rumor is freely fictitious that I.nom every.acc recipe cook
	das ein Koch, den ich aus dem Fernsehen kenne, kreiert.
	that.acc a.nom chef who.acc I.nom from TV know creates
	‘The rumor is a complete fabrication that I cook every recipe that a cook that I know from TV creates.’
**HIGH PROCESSING LOAD**		
Center	Das Gerücht, dass der Sohn jedes Rezept, das ein Koch,
embedded	the.nom rumor that the.nom son every.acc recipe that.acc a.nom chef
	den der Vater aus dem Fernsehen kennt, kreiert, nachkocht, ist frei erfunden.
	who.acc the.nom father from TV know creates cooks is freely fictitious
	‘The rumor that the son cooks every recipe that a cook that the father knows from TV creates is a complete fabrication.’
Control	Das Gerücht ist frei erfunden, dass der Sohn jedes Rezept nachkocht,
	the.nom rumor is freely fictitious that the.nom son every.nom recipe cooks
	das ein Koch, den der Vater aus dem Fernsehen kennt, kreiert.
	that a.nom chef who.acc the.nom father from TV know creates
	‘The rumor is a complete fabrication that the son cooks every recipe that a cook that the father knows from TV creates.’

#### 5.1.3. Procedure

Acceptability was tested using a questionnaire in the same way as in the two preceeding experiments.

### 5.2. Results

**Figure 4:**
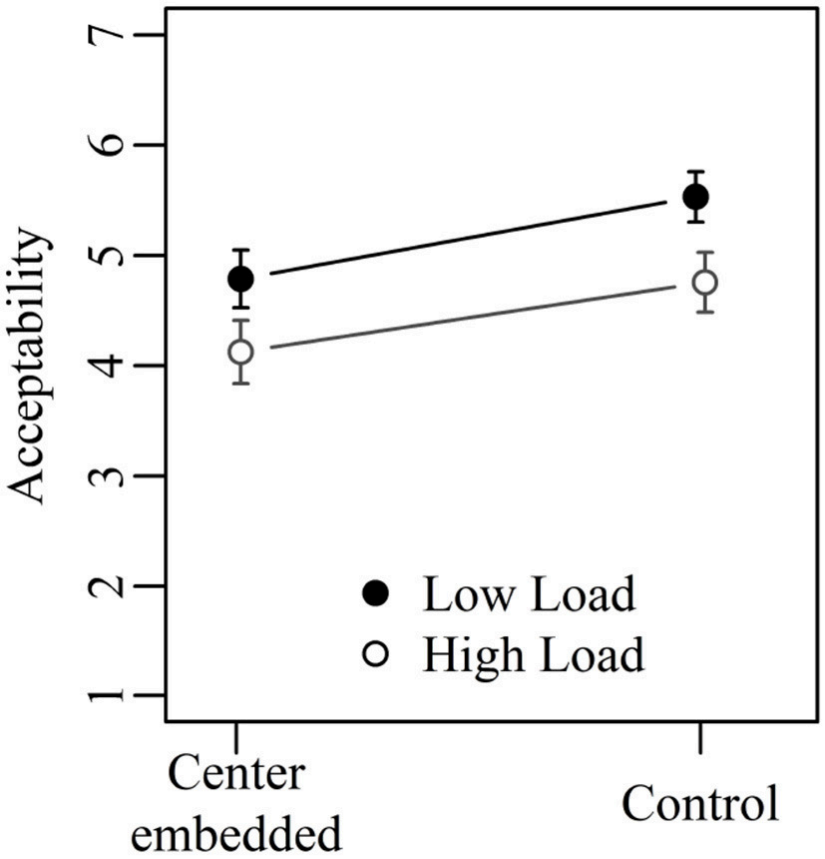
Mean acceptability ratings on a scale from 1 (low) to 7 (high) for Experiment 3. Error bars show 95% confidence intervals.

The data analysis proceeded as in the preceeding experiments. Figure 4 shows the mean acceptability ratings obtained in Experiment 3. The results of the linear mixed model fitted to the data are given in Table 9. The two main effects were significant but the interaction between them was not. Low-load sentence were judged as more acceptable than high-load sentences (5.2 vs. 4.4) and control sentences were judged as more acceptable than center-embedded sentences (5.1 vs. 4.5).

**Table 9:** Linear mixed model fitted by maximum likelihood estimation for Experiment 3, including p-values from likelihood ratio tests.

	**Coefficient**	**Std. Error**	***t*-value**	***p* (LRT)**
(Intercept)	4.8000	0.1503	31.94	
Structure	−0.6875	0.1904	−3.61	<0.001
Load	0.7188	0.1636	4.39	<0.01
Structure:load	−0.1125	0.2882	−0.39	>0.1

Acceptability ~ structure ^*^ load + (structure ^*^ load || subject) + (structure ^*^ load || sentence).

### 5.3. Discussion

Experiment 3 has yielded two major results. The first one is that sentences with lower integration cost were more acceptable than sentences with higher integration cost. Thus, when storage cost is held constant, effects of integration cost become visible. Furthermore, the effect of integration cost on acceptability was equal in size for center-embedded and for control sentences. This supports Gibson's (2000) hypothesis that acceptability ratings reflect maximum integration cost, since in both the center-embedded and the control condition, high- and low-load sentences differed by the same amount of two memory units when considering maximum integration cost. When considering summed integration cost, in contrast, the integration cost difference for center-embedded sentences was twice as high as for control sentences. This should have resulted in an interaction between structure and load, but no interaction was found.

The second major finding yielded by Experiment 3 is that even sentences with three degrees of center embedding were far from total unacceptability. In the low-load condition, center-embedded sentences received a mean acceptability rating of 4.8, which is well above the midpoint of the 1-to-7 scale. In the high-load condition, mean acceptability was 4.1 for center-embedded sentences, and thus almost exactly at the midpoint. One reason for this relative high acceptability despite three levels of center embedding may be that the highest center-embedded clause was a complement clause and not a relative clause. That clause type matters in configurations of multiple center embedding has been shown by Chen et al. (2005), who found that processing is easier when a complement clause contains a relative clause than when a relative clause contains a further relative clause (see also Gibson, 1998).

## 6. General discussion: syntactic dependencies and sentence complexity

This paper has presented three acceptability experiments investigating processing complexity in sentences with multiply nested dependencies. Experiment 1 compared sentences with three nested dependencies all originating in a single 3-verb cluster to sentences with four nested dependencies originating in two separate 2-verb clusters. The sentences with three nested dependencies were found to be substantially less acceptable than the sentences with four nested dependencies. This falsifies De Vries et al.'s (2011) generalization that sentences with three or more nested dependencies are difficult or even impossible to process by the human parsing mechanism.

Experiment 2 and Experiment 3 explored alternative sources of sentence complexity. Experiment 2 investigated sentences for which integration and storage cost lead to opposing predictions. The results of Experiment 2 confirmed the predictions from storage cost and thereby showed that storage cost outweighs integration cost as predictor of sentence complexity. Experiment 3 finally showed that integration cost still has an influence on sentence complexity when comparing sentences of equal storage cost.

The experimental findings are supported by an ongoing corpus study that searched for sentences with complex verb clusters in the deWaC corpus, a large corpus of written internet texts (Baroni et al., 2009). The deWac corpus has been annotated for lemma and part of speech information, but it is not a treebank. It was therefore not possible to retrieve sentences by searching for particular syntactic structures. Instead, the search had to proceed by specifying strings of tokens constrained by lexical information. In order not to miss relevant examples, the search string had to be specified rather loosely, making it necessary to remove irrelevant sentences by hand. For that reason, quantitative information is not yet available for the structures under consideration, although certain tendencies are discernible.

A large number of sentences with verb clusters containing at least three verbs were found, but none of the types investigated by Bach et al. (1986) and in Experiment 1. Instead, complex clusters either contained at most two argument-taking verbs plus additional non-argument-taking verbs (auxiliaries, modals). Sentences with 3 or more nested dependencies distributed across two separate verb clusters were found, however, as shown in Table 10. Overall, such examples are rare, but this is not unexpected because they must contain subpatterns that are themselves not very frequent, namely an embedded clause that has not been extraposed, and at least one verb cluster with two argument-taking verbs. Crucially, examples with three or more nested dependencies do occur, and they are not particularly difficult to comprehend, in accordance with the experimental results yielded by the preceding experiments.

**Table 10:** Authentic examples from the deWaC corpus (Baroni et al., 2009) with 3 or 4 nested dependencies.

**3 NESTED DEPENDENCIES: 2 UPPER, 1 LOWER**
Die Natur erspart den Wissenschaftlern derartige Reisen, indem sie Bruchstücke von Asteroiden, die aus irgendwelchen Gründen **zerborsten sind**, als Meteoriten zur Erde **herabregnen läßt**.
‘Nature spares scientists such journeys because it **lets** debris of asteroids **rain down** as meteorites that **bursted** for some reason.’
**3 NESTED DEPENDENCIES: 1 UPPER, 2 LOWER**
Auch wenn jeder, der einmal die dramatische Baumasse Manhattans aus dem Meer **wachsen sah**, größte Schwierigkeiten **haben dürfte**, sich an diesem Ort Wälder, Hügel, Wiesen und Marschen vorzustellen.
‘Even if anyone who **saw** the massive bulk of buildings of Manhattan **growing out** of the sea **might have** difficulties imagining woods, hills, meadows and marsh at this place.’
**4 NESTED DEPENDENCIES: 2 UPPER, 2 LOWER**
Sie erkannten, dass sie zuerst einmal die Kultur des jeweiligen Landes, das sie **zu missionieren beabsichtigten, kennen- und schätzen lernen mussten** und nicht mit einer gewissen europäischen Arroganz die dortigen Gepflogenheiten sofort als “Teufelswerk” ablehnen sollten.
‘They recognized that first, they should **try to get to know and appreciate** the culture of the country they **are aiming to evangelize** and that they should not reject local customs as a creation of the devil.’

Before going on, it should be pointed out that sentences that are easy to comprehend despite containing three or more nested dependencies are nothing special about German. Relevant examples may be somewhat easier to construct in a verb-final language, but they can also be found in an SVO language like English, as shown by the examples in (21) and (22).

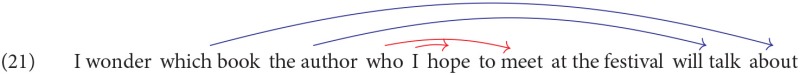

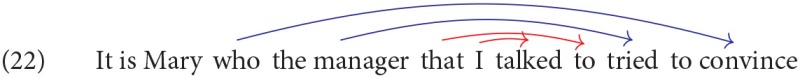

(21) and (22) both contain only a single level of center embedding, but nevertheless four nested dependencies, two in the upper clause and two in the lower clause. Thus, as in German, increasing the number of nested dependencies without increasing the degree of center embedding makes English sentences not overly hard to process.

The major question pursued in this paper concerned the role of dependency formation in accounting for syntactic complexity. With regard to this question, the results yielded by Experiments 1–3 indicate that the number of nested dependencies that a sentence contains is a poor predictor of sentence complexity. Thus, the Incomplete Dependency Hypothesis in (3) is invalid for dependencies independently of their order. In contrast to the number of dependencies, dependency length, as captured in terms of integration cost, was found to affect sentence complexity. Importantly, however, storage cost, a measure not related to dependency formation but to phrase-structure building, turned out to be more important than integration cost, which had an effect only when storage cost was held constant.

**Figure 5:**
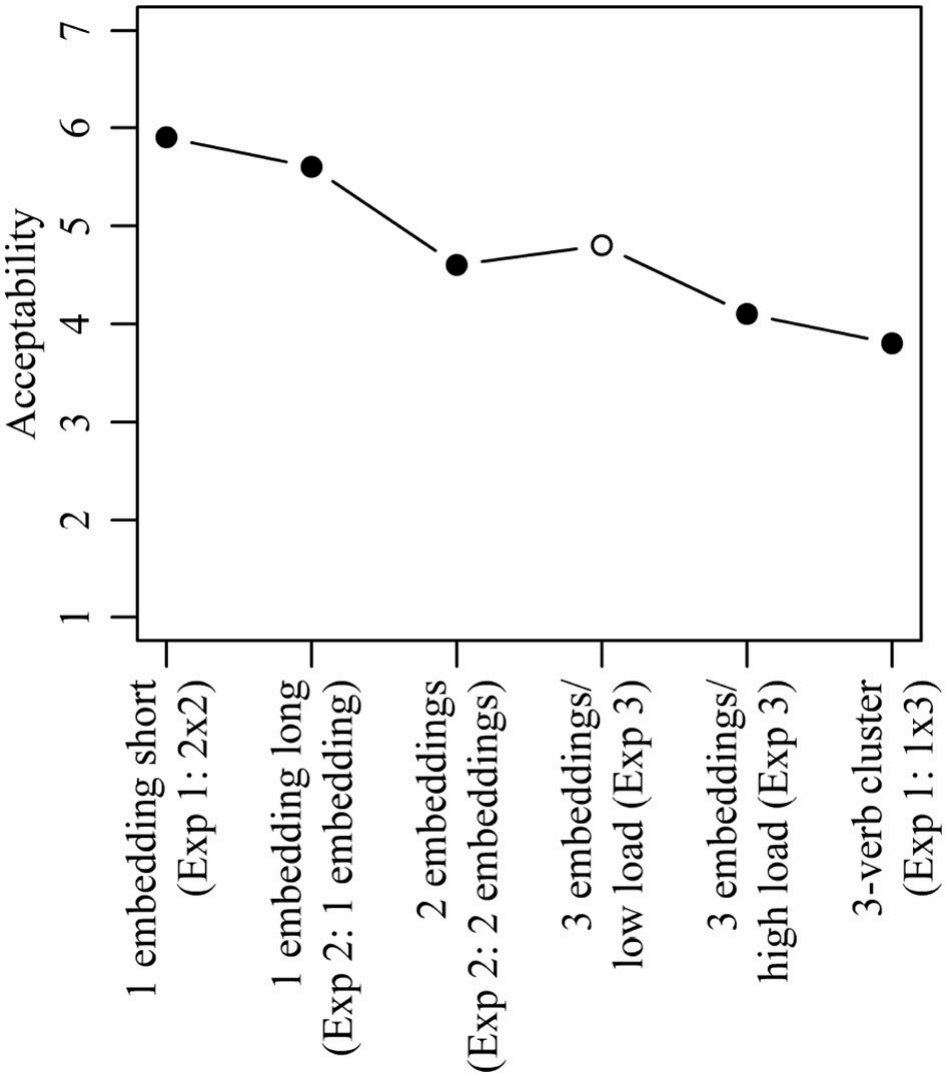
Overview of relationship between degree of center embedding and acceptability as found in Experiments 1–3. The experiment from which the data point has been taken is given in parenthesis. Filled circles are conditions with lexical NPs only; the open circle is the condition with pronominal NPs instead of lexical NPs.

Storage cost was measured by the number of predicted verbal heads. Because each clause obligatorily contains a verbal head, storage costs measured in this way directly reflects degree of center embedding. One degree of center embedding is associated with a maximal storage cost of two verbal heads, two degrees of center embedding are associated with a maximal storage cost of three verbal heads, and so on. To explore the relationship between storage cost/degree of center embedding and acceptability in more detail, Figure 5 provides a graphical summary of the results obtained in the preceding experiments.

Figure 5 shows that acceptability decreases with each additional step of center embedding as long as only sentences with full NPs are considered. The decrease in acceptability is modest at each step, and sentences with three degrees of center embedding are still above the midpoint of the 1-to-7 scale. About the same value was observed for sentences with 3-verb clusters as investigated in Experiment 1. We can therefore conclude that at least up to a degree of three center embeddings, acceptability decreases in a gradient fashion. If the trend visible in Figure 5 continues, sentences with still further levels of center embedding will become more and more unacceptable. A further finding visible in Figure 5 is that sentences with three degrees of center embedding and pronominal NPs in place of some of the full NPs were as acceptable as sentences with only two degrees of center embedding but with full NPs throughout. In sum, the degree of center embedding seems to be an important, or perhaps even the most important, predictor of acceptability, but its influence can be modulated by other factors.

The finding that acceptability degrades gracefully with increasing degree of center embedding makes it unlikely that a categorical limit on center embedding can be found, a limit that cleanly separates acceptable from unacceptable embedding. This argues against theories that ascribe the severe limitation on center embedding to the existence of a memory system that provides only a small, fixed amount of storage space for processing center embedded sentences (e.g., Yngve, 1960; Kimball, 1975; Stabler, 1994). Instead, this graceful degradation argues in favor of a multi-factorial account of the limits on center embedding. Two factors affecting sentence complexity are storage cost and integration cost, as shown by the experiments reported above. Other general factors that have been invoked to explain sentence complexity are frequency (e.g., Hale, 2011) and interference (e.g., Van Dyke and McElree, 2006, Belletti and Rizzi, 2013). Furthermore, for the case of sentences with multiple center embedding, Fodor (2013) has proposed that parsing difficulties arise because of difficulties with assigning a prosodic structure to such sentences. For reasons of space, it must be left as a task for future research to determine how the complexity of syntactic parsing follows from the joint work of the various factors.

To conclude, the results reported in this paper add to the existing evidence that the sheer number of open dependencies is not a crucial factor determining sentence complexity, independently of the the order of the dependencies. It is true that in many cases, more complex sentences contain more nested dependencies, but such sentences are typically also more complex in other ways. For example, sentences with doubly center-embedded relative clauses usually contain more nested dependencies than sentences with only a single degree of embedding, but they are also more complex in terms of storage cost, for example.

## Author contributions

The author confirms being the sole contributor of this work and approved it for publication.

### Conflict of interest statement

The author declares that the research was conducted in the absence of any commercial or financial relationships that could be construed as a potential conflict of interest.

## Footnotes

^1^For reasons of simplicity, the phrase-structure trees in (7) and (8) are expressed in terms of $\bar{\text{S}}$, S and VP. Using a more articulated structure involving, for example, CP, IP, and VP, wouldn't change the argument.
