# Supplementary material for: The Limited Role of Number of Nested Syntactic Dependencies in Accounting for Processing Cost: Evidence from German Simplex and Complex Verbal Clusters
Source: Front Psychol. 2018 Jan 23;8:2268. doi: 10.3389/fpsyg.2017.02268 (PMC5787339; doi:10.3389/fpsyg.2017.02268)
Supplement: Supplementary file 1 [file Presentation1.pdf]

frontiers

Supplementary Material: Sentence Complexity, Syntactic Dependencies, and Verb Cluster Formation

Markus Bader*
*Correspondence:
Markus Bader
bader@em.uni-frankfurt.de

This supplemental material contains three parts:

- A detailed discussion of integration and storage cost in sentences with complex verb clusters.
- The instruction of the questionnaires used to obtain acceptability judgments.
- The experimental sentences of Experiments 1 – 3.

1 INTEGRATION AND STORAGE COSTS FOR COMPLEX VERB CLUSTERS

This section shows that the basic findings concerning the parsing complexity of sentences with complex verb clusters do not follow from the integration cost or the storage cost associated with these sentences.

1.1 Integration cost of Dutch and German 3-verb clusters

In order to compute integration cost profiles for Dutch and German sentences with 3-verb clusters, it is necessary to specify the point in time at which each verb is integrated with its arguments. There are two basic options. Either each verb is integrated with its arguments as soon at it is encountered, or integration takes place when the verb cluster is complete, that is, after the last verb. Given the broad evidence for incremental syntactic parsing (Pickering and van Gompel, 2006), the first option is adopted in the following.

Under the assumption that each verb is integrated with its arguments as soon as the verb is encountered, the integration cost profile for a Dutch 3-verb cluster looks as in (1).

(1) Dutch sentences with one 3-verb cluster: crossed dependencies

	dass	Jan	Piet	Marie	zag	laten	zwemmen.
Referential cost		1	1	1	1	1	1
Structural cost		0	0	0	2	2+0	2+0
Total cost		1	1	1	3	3	3

Each NP and each verb is associated with a referential integration cost of one unit. The NPs are all associated with zero structural integration cost because the verbs with which they must integrate have not yet been encountered. Each verb is associated with a constant structural integration cost of two units. For example, integrating *zag* (‘saw’) with its subject NP *Jan* spans the two discourse referents introduced by *Piet* and *Marie*. The second and the third verb must additionally integrate with the verb by which they are selected. Because this is the directly preceding verb in both cases, zero structural integration costs ensue.

The assumption that argument linking proceeds incrementally gives the integration cost profile for a German 3-verb cluster sentence in (2).

(2) German sentences with one 3-verb cluster: nested dependencies

	dass	Jan	Piet	Marie	schwimmen	lassen	sah.
Referential cost		1	1	1	1	1	1
Structural cost		0	0	0	0	2+0	4+0
Total cost		1	1	1	1	3	5

The initial verb in the verb-cluster does not incur structural integration costs because nothing intervenes between it and its dependent NP. The second verb incurs a processing cost of 2 units because two new referents have been introduced between *lassen* ('let') and *Piet*, namely the referents of *Marie* and *schwimmen* ('swim'). The additional integration with its verbal complement *schwimmen* does not cause integration costs due to the adjacency of the two verbs. Similar considerations show that structural integration cost is 4 units at the final verb.

In sum, due to the nested dependency order of German, structural integration cost increases from zero for the first verb to four for the last verb. For Dutch with its crossed dependencies, in contrast, each verb is associated with the same structural integration cost of two. If we follow Gibson (2000) and make the assumption that sentence acceptability reflects the maximal integration cost observed at any point within a sentence, we get an account of Bach et al.'s finding that German nested dependencies are somewhat less acceptable than Dutch crossed dependencies.

1.2 Integration cost for the 2×2 sentences of Experiment 1

The integration cost profile for the 2×2 sentences of Experiment 1 is given in (3). As for the case of German 3-verb clusters discussed above, integration cost increases at each verb and reaches its highest value at the last verb (*sah* 'saw'). This verb has to be integrated with the initial argument *Moritz*. Because two new nominal referents and three verbal arguments were introduced between *Moritz* and *sah*, a structural integration cost of 5 results.

(3) 2×2 sentence of Experiment 1

	dass	Moritz	den Gärtner,	der	den Kunden	warten	ließ,	arbeiten	sah.
Referential cost		1	1	0	1	1	1	1	1
Structural cost		0	0	0	0	0	2	3	5
Total cost		1	1	0	1	1	3	4	6

Integration cost is greater in 2×2 sentences than in 1×3 sentences, both with regard to maximal integration cost and with regard to the total amount of integration cost. Integration cost therefore does not provide an account of the experimental findings of Experiment 1.[1]

In sum, the memory requirements posed by the need to form syntactic dependencies do not give us an account of why certain verb clusters with 3 or more verbs are hard to process for the human parser. Since the number of open dependencies also does not make the correct predictions for Experiment 1, we have to

[1] Lewis (1996) has proposed that it is not the total number of open dependencies that counts, but the number of dependencies of equal type. The conclusion reached in the main text also holds under this more specific proposal. 2×2 sentences contain two dependencies between a verb and its subject and two dependencies between a verb and its accusative object. 1×3 sentences contain one dependency between verb and subject and 2 dependencies between verb and accusative object. Thus, this measure also predicts that 2×2 sentences should be more complex than 1×3 sentences, contrary to the findings of Experiment 1.

look for alternatives to dependency formation in order to understand why sentences with certain types of verb clusters are difficult to process.

1.3 Storage cost for the for 1×3 and 2×2 sentences of Experiment 1

For 2×2 sentences, the parser has to predict one verbal head after encountering the sentence initial complementizer *dass* ('that'). Since a single verb can accommodate a subject and an accusative object, storage cost does not increase when processing the two argument NPs following the complementizer. On encountering the relative pronoun following the head noun of the accusative object, a second verbal head must be predicted, resulting in a storage cost of two memory units. This is already the maximum storage cost for 2×2 sentences, because no further verbal head has to be predicted at any later point.

Computing storage cost for 1×3 sentences is somewhat more involved. As before, one verbal head must be predicted on encountering the clause-initial complementizer. Since many verbs have a subject and an accusative object, no further predictions are necessary when processing the first two arguments. With the third argument, the parser finds a second accusative object. Verbs with two accusative objects exist in German, but their number is extremely small (< 10), they occur with low frequency, and they normally do not allow two animate objects. Thus, in principle, the parser could stick to the prediction of a single verb after encountering the second accusative object. However, it is more likely that the parser predicts a second verbal head at this point, in a way similar to when it encounters a second nominative marked NP in Japanese (cf. Nakatani and Gibson, 2009). This corresponds to a sentence as in (4), where each accusative object depends on a different verb.

(4) … dass Moritz den Gärtner den Kunden bedienen sah.
that M. the gardener the costumer serve saw
'…that Moritz saw the gardener serve the costumer.'

Under the assumption that the parser predicts a second verb when encountering the second accusative NP, storage cost increases to two memory units at this point of the parse. The predicted verbs are encountered next and so the maximal storage cost is two memory units. Thus, the storage cost of 1×3 sentences is not higher than the storage cost for 2×2 sentences. The finding that 1×3 sentences are much less acceptable than 2×2 sentences can accordingly not be explained in terms of storage cost.

REFERENCES

Bach, E., Brown, C., and Marslen-Wilson, W. D. (1986). Crossed and nested dependencies in German and Dutch: A psycholinguistic study. *Language and Cognitive Processes* 1, 249–262

Gibson, E. (2000). The dependency locality theory: A distance-based theory of linguistic complexity. In *Image, language, brain. Papers from the first Mind Articulation Project Symposium*, eds. A. Marantz, Y. Miyashita, and W. O'Neil (Cambridge, MA: MIT Press). 95–126

Lewis, R. L. (1996). Interference in short-term memory: The magical number two (or three) in sentence processing. *Journal of Psycholinguistic Research* 25, 93–115

Nakatani, K. and Gibson, E. (2009). An on-line study of Japanese nesting complexity. *Cognitive Science* 33, 1–19

Pickering, M. J. and van Gompel, R. P. G. (2006). Syntactic parsing. In *Handbook of Psycholinguistics*, eds. M. Traxler and M. Gernsbacher (New York: Academic Press). 2 edn., 455–503

2 INSTRUCTIONS FOR PARTICIPANTS

Ihre Aufgabe ist es, die Akzeptabilität von Sätzen auf einer Skala von 1 (völlig unakzeptabel) bis 7 (völlig akzeptabel) zu bewerten. Völlig akzeptabel ist ein Satz dann, wenn an ihm nichts auszusetzen ist. Völlig unakzeptabel ist ein Satz dagegen, wenn er so fehlerhaft ist, dass er in der Form niemals vorkommen würde.

Gehen Sie bei Ihrer Bewertung ausschließlich nach ihrem Gefühl vor und denken Sie nicht darüber nach, was man Ihnen in der Schule oder sonstwo über "gutes" oder "schlechtes" Deutsch erzählt hat. Es zählt ausschließlich Ihre persönliche Intuition!

'Your task is to rate the acceptability of sentences on a scale from 1 (completely unacceptable) to 7 (completely acceptable). A sentence is completely acceptable if you do not see any faults. A sentence is completely unacceptable, if it is too flawed to be used by anyone.

Rate all sentences exclusively by instinct and do not take into account what you were told in school concerning "good" or "bad" German. Only your personal intuition counts.'

3 EXPERIMENTAL SENTENCES

3.1 The experimental sentences of Experiment 1

The following are the experimental sentences of Experiment 1. For the condition “center-embedded”, the 1×3 version of each sentence is given in (a), the 2×2 version is given in (b). The corresponding control sentences are given in (a’) and (b’). An experimental sentence and its control sentence always have the same meaning, a translation is therefore only provided for the experimental sentence.

(1) a. Ich weiß, dass Peter den Moderator den Sänger auftreten lassen sah.
‘I know that Peter saw the host letting the singer perform.’
b. Ich weiß, dass Peter den Moderator, der den Sänger auftreten ließ, grinsen sah.
‘I know that Peter saw the host who let the singer perform grin.’
a’ Ich weiß, dass Peter sah, wie der Moderator den Sänger auftreten ließ.
b’ Ich weiß, dass Peter den Moderator grinsen sah, der den Sänger auftreten ließ.

(2) a. Ich weiß, dass Moritz den Gärtner den Kunden warten lassen sah.
‘I know that Moritz saw the gardener letting the customer wait.’
b. Ich weiß, dass Moritz den Gärtner, der den Kunden warten ließ, arbeiten sah.
‘I know that Moritz saw the gardener who let the customer wait work’
a’ Ich weiß, dass Moritz sah, wie der Gärtner den Kunden warten ließ.
b’ Ich weiß, dass Moritz den Gärtner arbeiten sah, der den Kunden warten ließ.

(3) a. Ich weiß, dass Klaus den Trainer den Sportler schwitzen lassen sah.
‘I know that Klaus saw the coach letting the athlete sweat.’
b. Ich weiß, dass Klaus den Trainer, der den Sportler schwitzen ließ, joggen sah.
‘I know that Klaus saw the coach who let the athlete sweat jog.’
a’ Ich weiß, dass Klaus sah, wie der Trainer den Sportler schwitzen ließ.
b’ Ich weiß, dass Klaus den Trainer joggen sah, der den Sportler schwitzen ließ.

(4) a. Ich weiß, dass Michael den Lehrer den Schüler nachsitzen lassen sah.
‘I know that Michael saw the teacher putting the pupil in detention.’
b. Ich weiß, dass Michael den Lehrer, der den Schüler nachsitzen ließ, telefonieren sah.
‘I know that Michael saw the teacher who put the pupil in detention telephone.’
a’ Ich weiß, dass Michael sah, wie der Lehrer den Schüler nachsitzen ließ.
b’ Ich weiß, dass Michael den Lehrer telefonieren sah, der den Schüler nachsitzen ließ.

(5) a. Ich weiß, dass Werner den Kommissar den Entführer verhören lassen sah.
‘I know that Werner saw the commissar getting the kidnapper interrogated’
b. Ich weiß, dass Werner den Kommissar, der den Entführer verhören ließ, rauchen sah.
‘I know that Werner saw the commissar who got the kidnapper interrogated smoke.’
a’ Ich weiß, dass Werner sah, wie der Kommissar den Entführer verhören ließ.
b’ Ich weiß, dass Werner den Kommissar rauchen sah, der den Entführer verhören ließ.

(6) a. Ich weiß, dass Kai den Polizeichef den Reporter abführen lassen sah.
‘I know that Kai saw the chief of police getting the reporter discharged.’
b. Ich weiß, dass Kai den Polizeichef, der den Reporter abführen ließ, gestikulieren sah.
‘I know that Kai saw the chief of police who got the reporter discharged gesticulate.’

a’ Ich weiß, dass Kai sah, wie der Polizeichef den Reporter abführen ließ.
b’ Ich weiß, dass Kai den Polizeichef gestikulieren sah, der den Reporter abführen ließ.

(7) a. Ich weiß, dass David den Reiseführer den Touristen bewirten lassen sah.
‘I know that David saw the tour guide getting the tourists entertained.’
b. Ich weiß, dass David den Reiseführer, der den Touristen bewirten ließ, trinken sah.
‘I know that David saw the tour guide who got the tourists entertained drink.’
a’ Ich weiß, dass David sah, wie der Reiseführer den Touristen bewirten ließ.
b’ Ich weiß, dass David den Reiseführer trinken sah, der den Touristen bewirten ließ.

(8) a. Ich weiß, dass Alexander den König den Dieb bestrafen lassen sah.
‘I know that Alexander saw the king letting the thief get punished.’
b. Ich weiß, dass Alexander den König, der den Dieb bestrafen ließ, lachen sah.
‘I know that Alexander saw the king who got the thief punished laugh.’
a’ Ich weiß, dass Alexander sah, wie der König den Dieb bestrafen ließ.
b’ Ich weiß, dass Alexander den König lachen sah, der den Dieb bestrafen ließ.

(9) a. Ich weiß, dass Peter den Einbrecher den Tresor aufzubrechen versuchen sah.
‘I know that Peter saw the burglar trying to break the safe.’
b. Ich weiß, dass Peter den Einbrecher, der den Tresor aufzubrechen versuchte, zittern sah.
‘I know that Peter saw the burglar who tried to break the safe tremble.’
a’ Ich weiß, dass Peter sah, wie der Einbrecher den Tresor aufzubrechen versuchte.
b’ Ich weiß, dass Peter den Einbrecher zittern sah, der den Tresor aufzubrechen versuchte.

(10) a. Ich weiß, dass Peter den Koch den Brand zu löschen versuchen sah.
‘I know that Peter saw the cook trying to put out the fire.’
b. Ich weiß, dass Peter den Koch, der den Brand zu löschen versuchte, verzweifeln sah.
‘I know that Peter saw the cook who tried to put out the fire despair.’
a’ Ich weiß, dass Peter sah, wie der Koch den Brand zu löschen versuchte.
b’ Ich weiß, dass Peter den Koch verzweifeln sah, der den Brand zu löschen versuchte.

(11) a. Ich weiß, dass Roland den Bergsteiger den Felsen zu erklimmen versuchen sah.
‘I know that Roland saw the mountaineer trying to climb the rock.’
b. Ich weiß, dass Roland den Bergsteiger, der den Felsen zu erklimmen versuchte, abstürzen sah.
‘I know that Roland saw the mountaineer who tried to climb the rock fall.’
a’ Ich weiß, dass Roland sah, wie der Bergsteiger den Felsen zu erklimmen versuchte.
b’ Ich weiß, dass Roland den Bergsteiger abstürzen sah, der den Felsen zu erklimmen versuchte.

(12) a. Ich weiß, dass Martin den Biologen den Baum zu retten versuchen sah.
‘I know that Martin saw the biologist trying to save the tree.’
b. Ich weiß, dass Martin den Biologen, der den Baum zu retten versuchte, beten sah.
‘I know that Martin saw the biologist who tried to save the tree pray.’
a’ Ich weiß, dass Martin sah, wie der Biologen den Baum zu retten versuchte.
b’ Ich weiß, dass Martin den Biologen beten sah, der den Baum zu retten versuchte.

(13) a. Ich weiß, dass Stefan den Zoodirektor den Elefanten zu beruhigen versuchen sah.
‘I know that Stefan saw the zoo director trying to soothe the elephant.’

b. Ich weiß, dass Stefan den Zoodirektor, der den Elefanten zu beruhigen versuchte, fluchen sah.
   'I know that Stefan saw the zoo director who tried to soothe the elephant curse.'
a' Ich weiß, dass Stefan sah, wie der Zoodirektor den Elefanten zu beruhigen versuchte.
b' Ich weiß, dass Stefan den Zoodirektor fluchen sah, der den Elefanten zu beruhigen versuchte.

(14) a. Ich weiß, dass Günther den Torhüter den Elfmeter abzuwehren versuchen sah.
   'I know that Günther saw the goalkeeper trying to parry the penalty kick off.'
b. Ich weiß, dass Günther den Torhüter, der den Elfmeter abzuwehren versuchte, jubeln sah.
   'I know that Günther saw the goalkeeper who tried to parry the penalty kick off cheer.'
a' Ich weiß, dass Günther sah, wie der Torhüter den Elfmeter abzuwehren versuchte.
b' Ich weiß, dass Günther den Torhüter jubeln sah, der den Elfmeter abzuwehren versuchte.

(15) a. Ich weiß, dass Harald den Zauberer den Knoten zu lösen versuchen sah.
   'I know that Harald saw the magician trying to untie the knot.'
b. Ich weiß, dass Harald den Zauberer, der den Knoten zu lösen versuchte, stolpern sah.
   'I know that Harald saw the magician who tried to untie the knot stumble.'
a' Ich weiß, dass Harald sah, wie der Zauberer den Knoten zu lösen versuchte.
b' Ich weiß, dass Harald den Zauberer stolpern sah, der den Knoten zu lösen versuchte.

(16) a. Ich weiß, dass Mario den Reiter den Steilhang zu umgehen versuchen sah.
   'I know that Mario saw the rider trying to avoid the steep slope.'
b. Ich weiß, dass Mario den Reiter, der den Steilhang zu umgehen versuchte, stürzen sah.
   'I know that Mario saw the rider who tried to avoid the steep slope fall.'
a' Ich weiß, dass Mario sah, wie der Reiter den Steilhang zu umgehen versuchte.
b' Ich weiß, dass Mario den Reiter stürzen sah, der den Steilhang zu umgehen versuchte.

3.2 The experimental sentences of Experiment 2

The following are the experimental sentences of Experiment 2. For the condition "center-embedded", the 1-embedding version of each sentence is given in (a), the 2-embedding version is given in (b). The corresponding control sentences are given in (a') and (b'). An experimental sentence and its control sentence always have the same meaning, a translation is therefore only provided for the experimental sentence.

(1) a. Ich weiß, dass Peter die Behauptung, dass der Moderator den Sänger auftreten ließ und dann kündigte, zu entkräften versuchte.
   'I know that Peter tried to refute the claim that the host let the singer perform and then resigned.'
b. Ich weiß, dass Peter die Behauptung, dass der Moderator, nachdem der Sänger aufgetreten war, kündigte, zu entkräften versuchte.
   'I know that Peter tried to refute the claim that the host resigned after the singer performed.'
a' Ich weiß, dass Peter die Behauptung zu entkräften versuchte, dass der Moderator den Sänger auftreten ließ und dann kündigte.
b' Ich weiß, dass Peter die Behauptung zu entkräften versuchte, dass der Moderator, nachdem der Sänger aufgetreten war, kündigte.

(2) a. Ich weiß, dass Moritz das Gerücht, dass der Architekt den Gärtner warten ließ und stattdessen frühstückte, zu verbreiten versuchte.

‘I know that Moritz tried to disseminate the rumour that the architect let the gardener wait and instead had breakfast.’

b. Ich weiß, dass Moritz das Gerücht, dass der Architekt, während der Gärtner gewartet hat, frühstückte, zu verbreiten versuchte.
‘I know that Moritz disseminated the rumour that the architect had breakfast while the gardener waited.’

a’ Ich weiß, dass Moritz das Gerücht zu verbreiten versuchte, dass der Architekt den Gärtner warten ließ und stattdessen frühstückte.

b’ Ich weiß, dass Moritz das Gerücht zu verbreiten versuchte, dass der Architekt, während der Gärtner gewartet hat, frühstückte.

(3) a. Ich weiß, dass Klaus die Anschuldigung, dass der Trainer den Sportler schwitzen ließ und dabei kiffte, aufzuklären versuchte.
‘I know that Klaus tried to clarify the accusation that the coach let the athlete sweat and smoked pot at the same time.’

b. Ich weiß, dass Klaus die Anschuldigung, dass der Trainer, während der Sportler schwitzen musste, kiffte, aufzuklären versuchte.
‘I know that Klaus tried to clarify the accusation that the coach smoked pot while the athlete had to sweat.’

a’ Ich weiß, dass Klaus die Anschuldigung aufzuklären versuchte, dass der Trainer den Sportler schwitzen ließ und dabei kiffte.

b’ Ich weiß, dass Klaus die Anschuldigung aufzuklären versuchte, dass der Trainer, während der Sportler schwitzen musste, kiffte.

(4) a. Ich weiß, dass der Direktor die Vermutung, dass der Lehrer den Schüler nachsitzen ließ und dabei einschlief, zu überprüfen versuchte.
‘I know that the director tried to review the assumption that the teacher kept the pupils in detention and fell asleep at it.’

b. Ich weiß, dass der Direktor die Vermutung, dass der Lehrer, während der Schüler nachsitzen musste, einschlief, zu überprüfen versuchte.
‘I know that the director tried to review the assumption that the teacher fell asleep while the pupil had to be in detention.’

a’ Ich weiß, dass der Direktor die Vermutung zu überprüfen versuchte, dass der Lehrer den Schüler nachsitzen ließ und dabei einschlief.

b’ Ich weiß, dass der Direktor die Vermutung zu überprüfen versuchte, dass der Lehrer, während der Schüler nachsitzen musste, einschlief.

(5) a. Ich weiß, dass der Polizeipräsident die Behauptung, dass der Kommissar den Entführer entwischen ließ und dann untertauchte, zu unterdrücken versuchte.
‘I know that the Chief of Police tried to suppress the claim that the inspector let the kidnapper escape and then went underground.’

b. Ich weiß, dass der Polizeipräsident die Behauptung, dass der Kommissar, nachdem der Entführer entwischt war, untertauchte, zu unterdrücken versuchte.
‘I know that the Chief of Police tried to suppress the claim that the inspector went underground after the kidnapper escaped.’

a’ Ich weiß, dass der Polizeipräsident die Behauptung zu unterdrücken versuchte, dass der Kommissar den Entführer entwischen ließ und dann untertauchte.

b’ Ich weiß, dass der Polizeipräsident die Behauptung zu unterdrücken versuchte, dass der Kommissar, nachdem der Entführer entwischt war, untertauchte.

(6) a. Ich weiß, dass Kai das Gerücht, dass der Reporter den Praktikanten recherchieren ließ und stattdessen verreiste, zu verbreiten versuchte.
‘I know that Kai disseminated the rumour that the reporter let the intern do research and instead went on a journey.’

b. Ich weiß, dass Kai das Gerücht, dass der Reporter, während der Praktikant recherchieren musste, verreiste, zu verbreiten versuchte.
‘I know that Kai disseminated the rumour that the reporter went on a journey while the intern had to do research.’

a’ Ich weiß, dass Kai das Gerücht zu verbreiten versuchte, dass der Reporter den Praktikanten recherchieren ließ und stattdessen verreiste.

b’ Ich weiß, dass Kai das Gerücht zu verbreiten versuchte, dass der Reporter, während der Praktikant recherchieren musste, verreiste.

(7) a. Ich weiß, dass der Anwalt die Behauptung, dass der Reiseführer den Rentner tauchen ließ und währendessen einkaufte, zu bestätigen versuchte.
‘I know that the lawyer tried to confirm the claim that the guide let the retiree dive while he went shopping.’

b. Ich weiß, dass der Anwalt die Behauptung, dass der Reiseführer, während der Rentner getaucht ist, einkaufte, zu bestätigen versuchte.
‘I know that the lawyer tried to confirm the claim that the travel guide went shopping while the retiree was diving.’

a’ Ich weiß, dass der Anwalt die Behauptung zu bestätigen versuchte, dass der Reiseführer den Rentner tauchen ließ und währendessen einkaufte.

b’ Ich weiß, dass der Anwalt die Behauptung zu bestätigen versuchte, dass der Reiseführer, während der Rentner getaucht ist, einkaufte.

(8) a. Ich weiß, dass Alexander die Behauptung, dass der König den Minister regieren ließ und stattdessen feierte, zu widerlegen versuchte.
‘I know that Alexander tried to falsify the claim that the king let the minister reign and instead celebrated.’

b. Ich weiß, dass Alexander die Behauptung, dass der König, während der Minister regieren musste, feierte, zu widerlegen versuchte.
‘I know that Alexander tried to falsify the claim that the king celebrated while the minister had to reign.’

a’ Ich weiß, dass Alexander die Behauptung zu widerlegen versuchte, dass der König den Minister regieren ließ und stattdessen feierte.

b’ Ich weiß, dass Alexander die Behauptung zu widerlegen versuchte, dass der König, während der Minister regieren musste, feierte.

(9) a. Ich weiß, dass Peter die Vermutung, dass der Einbrecher den Tresor aufzubrechen versuchte und dabei zitterte, überprüfen ließ.

‘I know that Peter let check the assumption that the burglar tried to break the safe and trembled at the same time.’

b. Ich weiß, dass Peter die Vermutung, dass der Einbrecher, nachdem der Tresor aufgebrochen war, zitterte, überprüfen ließ.
   ‘I know that Peter tried to check the assumption that the burglar trembled after the safe was broken.’

a’ Ich weiß, dass Peter die Vermutung überprüfen ließ, dass der Einbrecher den Tresor aufzubrechen versuchte und dabei zitterte.

b’ Ich weiß, dass Peter die Vermutung überprüfen ließ, dass der Einbrecher, nachdem der Tresor aufgebrochen war, zitterte.

(10) a. Ich weiß, dass der Wirt die Behauptung, dass der Koch den Brand zu löschen versuchte und dann verzweifelte, verbieten ließ.
   ‘I know that the landlord got the claim banned that the cook tried to put out the fire and then despaired.’

b. Ich weiß, dass der Wirt die Behauptung, dass der Koch, nachdem der Brand gelöscht war, verzweifelte, verbieten ließ.
   ‘I know that the landlord got the claim banned that the cook despaired after the fire was put out.’

a’ Ich weiß, dass der Wirt die Behauptung verbieten ließ, dass der Koch den Brand zu löschen versuchte und dann verzweifelte.

b’ Ich weiß, dass der Wirt die Behauptung verbieten ließ, dass der Koch, nachdem der Brand gelöscht war, verzweifelte.

(11) a. Ich weiß, dass der Reporter das Gerücht, dass der Bergsteiger den Felsen zu erklimmen versuchte und dann abstürzte, überprüfen ließ.
   ‘I know that the reporter got the rumour checked that the mountaineer tried to climb the rock and then fell.’

b. Ich weiß, dass der Reporter das Gerücht, dass der Bergsteiger, nachdem der Felsen erklommen war, abstürzte, überprüfen ließ.
   ‘I know that the reporter got the rumour checked that the mountaineer fell after the rock had been climbed.’

a’ Ich weiß, dass der Reporter das Gerücht überprüfen ließ, dass der Bergsteiger den Felsen zu erklimmen versuchte und dann abstürzte.

b’ Ich weiß, dass der Reporter das Gerücht überprüfen ließ, dass der Bergsteiger, nachdem der Felsen erklommen war, abstürzte.

(12) a. Ich weiß, dass Martin die Mitteilung, dass der Aktivist den Baum zu retten versuchte und dann betete, verbreiten ließ.
   ‘I know that Martin got the message spread that the activist tried to save the tree and then prayed.’

b. Ich weiß, dass Martin die Mitteilung, dass der Aktivist, nachdem der Baum gerettet war, betete, verbreiten ließ.
   ‘I know that Martin tried to spread the message that the activist prayed after the tree was saved.’

a’ Ich weiß, dass Martin die Mitteilung verbreiten ließ, dass der Aktivist den Baum zu retten versuchte und dann betete.

b’ Ich weiß, dass Martin die Mitteilung verbreiten ließ, dass der Aktivist, nachdem der Baum gerettet war, betete.

(13) a. Ich weiß, dass der Zoodirektor die Behauptung, dass der Tierpfleger den Elefant zu beruhigen versuchte und dann zusammenbrach, bestätigen ließ.
‘I know that the zoo director got the claim confirmed that the zookeeper tried to soothe the elephant and then collapsed.’
b. Ich weiß, dass der Zoodirektor die Behauptung, dass der Tierpfleger, nachdem der Elefant beruhigt war, zusammenbrach, bestätigen ließ.
‘I know that the zoo director got the claim confirmed that the zookeeper collapsed after the elephant was soothed.’
a’ Ich weiß, dass der Zoodirektor die Behauptung bestätigen ließ, dass der Tierpfleger den Elefant zu beruhigen versuchte und dann zusammenbrach.
b’ Ich weiß, dass der Zoodirektor die Behauptung bestätigen ließ, dass der Tierpfleger, nachdem der Elefant beruhigt war, zusammenbrach.

(14) a. Ich weiß, dass der Trainer die Behauptung, dass der Torhüter den Elfmeter abzuwehren versuchte und dann ausrastete, dementieren ließ.
‘I know that the coach got the claim denied that the goalkeeper tried to parry the penalty kick and then freaked.’
b. Ich weiß, dass der Trainer die Behauptung, dass der Torhüter, nachdem der Elfmeter abgewehrt war, ausrastete, dementieren ließ.
‘I know that the coach got the claim denied that the goalkeeper freaked after the penalty kick was parried.’
a’ Ich weiß, dass der Trainer die Behauptung dementieren ließ, dass der Torhüter den Elfmeter abzuwehren versuchte und dann ausrastete.
b’ Ich weiß, dass der Trainer die Behauptung dementieren ließ, dass der Torhüter, nachdem der Elfmeter abgewehrt war, ausrastete.

(15) a. Ich habe gelesen, dass der Geheimdienst die Vermutung, dass der Informatiker den Geheimcode zu entziffern versuchte und dann verschwand, widerlegen ließ.
‘I read that the secret service got the assumption falsified that the computer scientist tried to decode the secret code and then disappeared.’
b. Ich habe gelesen, dass der Geheimdienst die Vermutung, dass der Informatiker, nachdem der Geheimcode entziffert war, verschwand, widerlegen ließ.
‘I read that the secret service got the assumption falsified that the computer scientist disappeared after the secret code was decoded.’
a’ Ich habe gelesen, dass der Geheimdienst die Vermutung widerlegen ließ, dass der Informatiker den Geheimcode zu entziffern versuchte und dann verschwand.
b’ Ich habe gelesen, dass der Geheimdienst die Vermutung widerlegen ließ, dass der Informatiker, nachdem der Geheimcode entziffert war, verschwand.

(16) a. Ich weiß, dass der Veranstalter die Behauptung, dass der Reiter den Steilhang zu umgehen versuchte und dann stürzte, dementieren ließ.
‘I know that the event organiser got the claim denied that the rider tried to avoid the steep slope and the fell.’
b. Ich weiß, dass der Veranstalter die Behauptung, dass der Reiter, nachdem der Steilhang umgangen war, stürzte, dementieren ließ.

‘I know that the event organiser got the claim denied that the rider fell after the steep slope was avoided.’

a’ Ich weiß, dass der Veranstalter die Behauptung dementieren ließ, dass der Reiter den Steilhang zu umgehen versuchte und dann stürzte.

b’ Ich weiß, dass der Veranstalter die Behauptung dementieren ließ, dass der Reiter, nachdem der Steilhang umgangen war, stürzte.

3.3 The experimental sentences of Experiment 3

The following are the experimental sentences of Experiment 3. For the condition “center-embedded”, the low-load version of each sentence is given in (a), the high-load version is given in (b). The corresponding control sentences are given in (a’) and (b’). An experimental sentence and its control sentence always have the same meaning, a translation is therefore only provided for the experimental sentence.

(1) a. Der Vorwurf, dass ich jeden Song, den ein Sänger, den ich nicht kenne, singt, ablehne, stimmt überhaupt nicht.
‘The accusation that I reject every song that a singer who I don’t know sings is not correct at all.’

b. Der Vorwurf, dass mein Kollege jeden Song, den ein Sänger, den der Chef nicht kennt, singt, ablehnt, stimmt überhaupt nicht.
‘The accusation that my colleague rejects every song that a singer who the boss doesn’t know sings is not correct at all.’

a’ Der Vorwurf, dass mein Kollege jeden Song, den ein Sänger, den der Chef nicht kennt, singt, ablehnt, stimmt überhaupt nicht.

b’ Der Vorwurf stimmt überhaupt nicht, dass mein Kollege jeden Song ablehnt, den ein Sänger, den der Chef nicht kennt, singt.

(2) a. Die Vermutung, dass ich jeden Vorschlag, den ein Politiker, mit dem ich verwandt bin, macht, unterstütze, ist nicht zu leugnen.
‘The assumption that I support any proposal which a politician with whom I am related makes cannot be denied.’

b. Die Vermutung, dass der Staatssekretär jeden Vorschlag, den ein Politiker, mit dem der Minister verwandt ist, macht, unterstützt, ist nicht zu leugnen.
‘The assumption that the state secretary supports any proposal which a politician with whom the secretary of state is related makes cannot be denied.’

a’ Die Vermutung, dass der Staatssekretär jeden Vorschlag, den ein Politiker, mit dem der Minister verwandt ist, macht, unterstützt, ist nicht zu leugnen.

b’ Die Vermutung ist nicht zu leugnen, dass der Staatssekretär jeden Vorschlag unterstützt, den ein Politiker, mit dem der Minister verwandt ist, macht.

(3) a. Das Gerücht, dass ich jedes Rezept, das ein Koch, den ich aus dem Fernsehen kenne, kreiert, nachkoche, ist frei erfunden.
‘The rumour that I cook any recipe that a cook who I know from TV creates is fictitious.’

b. Das Gerücht, dass der Sohn jedes Rezept, das ein Koch, den der Vater aus dem Fernsehen kennt, kreiert, nachkocht, ist frei erfunden.
‘The rumour that the sun cooks any recipe that a cook who the father knows from TV creates is fictitious.’

a’ Das Gerücht, dass der Sohn jedes Rezept, das ein Koch, den der Vater aus dem Fernsehen kennt, kreiert, nachkocht, ist frei erfunden.

b’ Das Gerücht ist frei erfunden, dass der Sohn jedes Rezept nachkocht, das ein Koch, den der Vater aus dem Fernsehen kennt, kreiert.

(4) a. Die Behauptung, dass ich kein Computerspiel, das ein Kollege, gegen den ich immer gewinne, empfohlen hat, spiele, ist vollkommen korrekt.
‘The claim that I don’t play any computer game that a colleague against whom I always win recommended is absolutely right.’

b. Die Behauptung, dass der Programmierer kein Computerspiel, das ein Kollege, gegen den der Projektleiter immer gewinnt, empfohlen hat, spielt, ist vollkommen korrekt.
‘The claim that a programmer doesn’t play any computer game that a colleague against whom the project leader always wins recommended is absolutely right.’

a’ Die Behauptung, dass der Programmierer kein Computerspiel, das ein Kollege, gegen den der Projektleiter immer gewinnt, empfohlen hat, spielt, ist vollkommen korrekt.

b’ Die Behauptung ist vollkommen korrekt, dass der Programmierer kein Computerspiel spielt, das ein Kollege, gegen den der Projektleiter immer gewinnt, empfohlen hat.

(5) a. Die Behauptung, dass ich jedes Buch, das von einem Autor, den ich gerne mag, geschrieben wurde, lese, stimmt überhaupt nicht.
‘The claim that I read any book that was written by an author who I love is not correct at all.’

b. Die Behauptung, dass der Klassenprimus jedes Buch, das von einem Autor, den der Lehrer gerne mag, geschrieben wurde, liest, stimmt überhaupt nicht.
‘The claim that the best student reads any book that was written by an author who the teacher loves is not correct at all.’

a’ Die Behauptung, dass der Klassenprimus jedes Buch, das von einem Autor, den der Lehrer gerne mag, geschrieben wurde, liest, stimmt überhaupt nicht.

b’ Die Behauptung stimmt überhaupt nicht, dass der Klassenprimus jedes Buch liest, das von einem Autor, den der Lehrer gerne mag, geschrieben wurde.

(6) a. Der Vorwurf, dass ich jedes Spiel, das von einem Schiedsrichter, den ich nicht leiden kann, gepfiffen wird, verpasse, ist völlig frei erfunden.
‘The accusation that I miss every match that is refereed by a referee who I dislike is fictitious.’

b. Der Vorwurf, dass der Mittelstürmer jedes Spiel, das von einem Schiedsrichter, den der Berater nicht leiden kann, gepfiffen wird, verpasst, ist völlig frei erfunden.
‘The accusation that the center-forward misses every match that is refereed by a referee who the advisor dislikes is fictitious.’

a’ Der Vorwurf, dass der Mittelstürmer jedes Spiel, das von einem Schiedsrichter, den der Berater nicht leiden kann, gepfiffen wird, verpasst, ist völlig frei erfunden.

b’ Der Vorwurf ist völlig frei erfunden, dass der Mittelstürmer jedes Spiel verpasst, das von einem Schiedsrichter, den der Berater nicht leiden kann, gepfiffen wird.

(7) a. Die Vermutung, dass ich jedes Album, das von einer Gruppe, die ich schon als Jugendlicher mochte, veröffentlich wurde, runterlade, liegt nicht ganz daneben.
‘The assumption that I download any album that was released by a group that I already loved as a teenager is not totally wrong.’

b. Die Vermutung, dass der Gitarrist jedes Album, das von einer Gruppe, die der Produzent schon als Jugendlicher mochte, veröffentlich wurde, runterlädt, liegt nicht ganz daneben.
‘The assumption that the guitarist downloads any album that was released by a group that the producer already loved as a teenager is not totally wrong.’
a’ Die Vermutung liegt nicht ganz daneben, dass ich jedes Album runterlade, das von einer Gruppe, die ich schon als Jugendlicher mochte, veröffentlich wurde.
b’ Die Vermutung liegt nicht ganz daneben, dass der Gitarrist jedes Album runterlädt, das von einer Gruppe, die der Produzent schon als Jugendlicher mochte, veröffentlich wurde.

(8) a. Das Gerücht, dass ich jedes Auto, das von einem Designer, den ich bewundere, entworfen wurde, kaufe, ist tatsächlich wahr.
‘The rumour that I buy every car that was created by a designer whom I admire is actually true.’
b. Das Gerücht, dass der Spieler jedes Auto, das von einem Designer, den der Trainer bewundert, entworfen wurde, kauft, ist tatsächlich wahr.
‘The rumour that the player buys every car that was created by a designer whom the team manager admires is actually true.’
a’ Das Gerücht ist tatsächlich wahr, dass ich jedes Auto kaufe, das von einem Designer, den ich bewundere, entworfen wurde.
b’ Das Gerücht ist tatsächlich wahr, dass der Spieler jedes Auto kauft, das von einem Designer, den der Trainer bewundert, entworfen wurde.

(9) a. Das Gerücht, dass ich kein Konzert, das ein Dirigent, von dem ich gefördert wurde, dirigiert, auslasse, ist tatsächlich wahr.
‘The rumour that I don’t miss any concert that a director by whom I was promoted conducts is actually true.’
b. Das Gerücht, dass der Startenor kein Konzert, das ein Dirigent, von dem der Widersacher gefördet wurde, dirigiert, auslässt, ist tatsächlich wahr.
‘The rumour that the famous tenor doesn’t miss any concert that a director by whom the antagonist was promoted conducts is actually true.’
a’ Das Gerücht ist tatsächlich wahr, dass ich kein Konzert auslasse, das ein Dirigent, von dem ich gefördet wurde, dirigiert.
b’ Das Gerücht ist tatsächlich wahr, dass der Startenor kein Konzert auslässt, das ein Dirigent, von dem der Widersacher gefördet wurde, dirigiert.

(10) a. Die Behauptung, dass ich jeden Unfug, den ein Mitschüler, mit dem ich gut auskomme, veranstaltet, mitmache, ist schon richtig.
‘The claim that I join in any mischief that a classmate with whom I get along well does is yet right.’
b. Die Behauptung, dass der Klassensprecher jeden Unfug, den ein Mitschüler, mit dem der Lehrer gut auskommt, veranstaltet, mitmacht, ist schon richtig.
‘The claim that class representative joins in any mischief that a classmate with whom the teacher gets along well does is yet right.’
a’ Die Behauptung ist schon richtig, dass ich jeden Unfug mitmache, den ein Mitschüler, mit dem ich gut auskomme, veranstaltet.
b’ Die Behauptung ist schon richtig, dass der Klassensprecher jeden Unfug mitmacht, den ein Mitschüler, mit dem der Lehrer gut auskommt, veranstaltet.

(11) a. Der Vorwurf, dass ich jedes Sonderangebot, das ein Kollege, mit dem ich schon mal zusammengearbeitet habe, entdeckt hat, kaufe, kann nicht bestritten werden.
'The accusation that I buy any special offer that a colleague with whom I already cooperated discovered can't be denied.'
b. Der Vorwurf, dass der Hausmeister jedes Sonderangebot, das ein Kollege, mit dem der Pförtner schon mal zusammengearbeitet hat, entdeckt hat, kauft, kann nicht bestritten werden.
'The accusation that the janitor buys any special offer that a colleague with whom the gatekeeper already cooperated discovered can't be denied.'
a' Der Vorwurf kann nicht bestritten werden, dass ich jedes Sonderangebot kaufe, das ein Kollege, mit dem ich schon mal zusammengearbeitet habe, entdeckt hat.
b' Der Vorwurf kann nicht bestritten werden, dass der Hausmeister jedes Sonderangebot kauft, das ein Kollege, mit dem der Pförtner schon mal zusammengearbeitet hat, entdeckt hat.

(12) a. Die Vermutung, dass ich jedes Angebot, das ein Manager, den ich nicht ausstehen kann, gemacht hat, ablehne, ist eine reine Erfindung.
'The assumption that I reject any offer that a manager who I hate made is pure fiction.'
b. Die Vermutung, dass der Boss jedes Angebot, das ein Manager, den der Eigentümer nicht ausstehen kann, gemacht hat, ablehnt, ist eine reine Erfindung.
'The assumption that the boss rejects any offer that a manager who the owner hates made is pure fiction.'
a' Die Vermutung ist eine reine Erfindung, dass ich jedes Angebot ablehne, das ein Manager, den ich nicht ausstehen kann, gemacht hat.
b' Die Vermutung ist eine reine Erfindung, dass der Boss jedes Angebot ablehnt, das ein Manager, den der Eigentümer nicht ausstehen kann, gemacht hat.

(13) a. Die Vermutung, dass ich jeden Fall, der von einem Staatsanwalt, mit dem ich persönlich bekannt bin, betreut wird, lösen kann, ist leider nicht wahr.
'The assumption that I can solve any case that is guided by a prosecutor with whom I am aquainted personally is unfortunately not true.'
b. Die Vermutung, dass der Kommissar jeden Fall, der von einem Staatsanwalt, mit dem der Assistent persönlich bekannt ist, betreut wird, lösen kann, ist leider nicht wahr.
'The assumption that the inspector can solve any case that is guided by a prosecutor with whom the assistant is aquainted personally is unfortunately not true.'
a' Die Vermutung ist leider nicht wahr, dass ich jeden Fall lösen kann, der von einem Staatsanwalt, mit dem ich persönlich bekannt bin, betreut wird.
b' Die Vermutung ist leider nicht wahr, dass der Kommissar jeden Fall lösen kann, der von einem Staatsanwalt, mit dem der Assistent persönlich bekannt ist, betreut wird.

(14) a. Das Gerücht, dass ich jeden Artikel, der von einem Journalisten, den ich verachte, verfasst wurde, kritisiere, ist eine gemeine Verleumdung.
'The rumour that I criticize any article that was written by a journalist whom I despise is a malicious falsehood.'
b. Das Gerücht, dass der Redakteur jeden Artikel, der von einem Journalisten, den der Ressortleiter verachtet, verfasst wurde, kritisiert, ist eine gemeine Verleumdung.
'The rumour that the editor criticizes any article that was written by a journalist whom the head of department despises is a malicious falsehood.'

a’ Das Gerücht ist eine gemeine Verleumdung, dass ich jeden Artikel kritisiere, der von einem Journalisten, den ich verachte, verfasst wurde.

b’ Das Gerücht ist eine gemeine Verleumdung, dass der Redakteur jeden Artikel kritisiert, der von einem Journalisten, den der Ressortleiter verachtet, verfasst wurde.

(15) a. Die Behauptung, dass ich jedes Gerücht, das von einem Informanten, den ich persönlich kenne, verbreitet wird, glaube, stimmt doch gar nicht.
‘The claim that I believe any rumour that is disseminated by an informant whom I know privately is absolutely not right yet.’

b. Die Behauptung, dass der Reporter jedes Gerücht, das von einem Informanten, den der Fotograf persönlich kennt, verbreitet wird, glaubt, stimmt doch gar nicht.
‘The claim that the reporter believes any rumour that is disseminated by an informant whom the photographer knows privately is absolutely not right yet.’

a’ Die Behauptung stimmt doch gar nicht, dass ich jedes Gerücht glaube, das von einem Informanten, den ich persönlich kenne, verbreitet wird.

b’ Die Behauptung stimmt doch gar nicht, dass der Reporter jedes Gerücht glaubt, das von einem Informanten, den der Fotograf persönlich kennt, verbreitet wird.

(16) a. Der Vorwurf, dass ich jeden Vortrag, der von einem Forscher, den ich beneide, gehalten wird, schwänze, ist völlig aus der Luft gegriffen.
‘The accusation that I skip any lecture that is given by a scientist who I envy is absolutely plucked out of the air.’

b. Der Vorwurf, dass der Doktorand jeden Vortrag, der von einem Forscher, den der Professor beneidet, gehalten wird, schwänzt, ist völlig aus der Luft gegriffen.
‘The accusation that the doctoral student skips any lecture that is given by a scientist who the professor envies is absolutely plucked out of the air.’

a’ Der Vorwurf ist völlig aus der Luft gegriffen, dass ich jeden Vortrag schwänze, der von einem Forscher, den ich beneide, gehalten wird.

b’ Der Vorwurf ist völlig aus der Luft gegriffen, dass der Doktorand jeden Vortrag schwänzt, der von einem Forscher, den der Professor beneidet, gehalten wird.
